# The Chemical Composition Characteristics and Health Risk Assessment of Cooking Fume Condensates from Residential Kitchens in Different Regions of China

**DOI:** 10.3390/foods12010106

**Published:** 2022-12-25

**Authors:** Qinghong Liu, Xiaofang Zhang, Yan Yang, Qiuxia Tang, Liting Zheng, Hongwei Lou, Huaguo Chen, Qin Yang

**Affiliations:** 1 College of Civil Engineering, Guizhou University, Guiyang 550025, China; 2 Guizhou Provincial Key Laboratory of Rock and Soil Mechanics and Engineering Safety, Guiyang 550025, China; 3 Guizhou Engineering Laboratory for Quality Control & Evaluation Technology of Medicine, Guizhou Normal University, Guiyang 550001, China

**Keywords:** residential kitchen, cooking fumes, volatile organic pollutants, chemical composition characteristics, health risk assessment

## Abstract

The aim of this study was to explore the similarities and differences of volatile organic pollutants (VOCs) in cooking fumes (COF) of residential buildings in different regions of China, as well as to evaluate their potential health risks. COF condensates were collected from 10 representative cities in China and analyzed by a GC–MS method. Their effects on α-glucosidase, acetylcholinesterase (AchE), and lactate dehydrogenase (LDH) activities were then detected to evaluate potential health risks. A total of 174 kinds of VOCs, including aldehydes, esters, hydrocarbons, alcohols, and carboxylic acid, were identified. There were 59 identical compounds in the northern and southern regions, and 56 common compounds in spicy and non-spicy regions. Health risk assessment results showed that COF condensate could inhibit the activity of α-glucosidase to varying degrees (61.73–129.25%), suggesting that it had a potential risk of causing hypoglycemia. Daily and 3 and 6 month intakes of COF in minors, adults, and the elderly had both activated and inhibited effects on AchE. The activated effect in the southern and spicy areas was higher than that in northern and non-spicy areas, revealing that different regions and dietary habits had different effects on the risk of neurological diseases caused by changes in AchE activity. For minors, adults, and the elderly, COF had different degrees of activation of LDH at different exposure times and regions. Activation in the northern and non-spicy areas was higher than that in southern and spicy areas, suggesting that the health risks caused by changes in LDH activity levels were significantly increased.

## 1. Introduction

The kitchen is an important indoor functional area where people spend considerable time on daily cooking [1]. It is a room with relatively poor indoor air quality and has great potential health risks [2]. Kitchen air pollution and harm mainly come from cooking fumes (COF) [3]. After the toxic and harmful components of COF enter the human body, they can generate a large number of reactive oxygen species (such as reactive oxide species, active methyl, etc.) through the metabolism of cytochrome P_450_ enzymes, resulting in the damage of biological macromolecules such as proteins, DNA, and lipids, as well as the disorder of normal physiological functions of cells [4]. COF also stimulate respiratory mucosa and lung cells, resulting in cytotoxic effects, increasing the risk of lung cancer [5]. Short-term exposure to a certain concentration of COF can easily cause respiratory irritation, chest tightness, shortness of breath, and other discomfort symptoms [6,7]. In addition, exposure to COF is also associated with fatty liver [8], diabetes [9], and increased risk of hyperactivity in pregnant women and their offspring [10]. Therefore, kitchen smoke pollution and its health risks have become one of the focuses of attention.

At present, the control of COF in the kitchen mostly adopts ventilation mode [11], such as the use of exhaust fan, exhaust hood, and range hood to directly discharge the kitchen fumes [12]. Although these methods can alleviate the degree of pollution of the kitchen indoor environment to some extent, they cannot essentially solve the problem of pollutants (COF) being discharged outdoors [13]. Therefore, an effective way to solve the problem of COF pollution is by clarifying the chemical substances in COF to their corresponding health risks and then carrying out targeted research on the degradation or elimination of pollutants.

As far as China is concerned, it has a vast territory with complex and diverse climatic environments. China can be divided into Northeast China, North China, Northwest China, Southwest China, Central China, East China, South China, and other administrative regions [14]. Different regions have long formed their own eating habits, resulting in unique cooking methods. For example, Tianjin in North China has strong tastes, and the cooking methods are mainly frying, boiling, roasting, and skimming. Sichuan Province in southwest China, with spicy fragrance as the main characteristic, is good with cooking methods such as stir-frying. At present, many articles have reported the association between COF exposure and disease risk in China. For example, Lu et al. [15] analyzed the concentration and composition of total volatile organic compounds in the respiratory zone under six different cooking methods (stir-frying, quick-frying, deep-frying, boiling, stewing, and steaming) by a GC–MS method and evaluated their potential health risks by mathematical models. Wei et al. [16] explored the relationship between COF and sleep quality in the middle-aged Chinese population. Yi et al. [17] reported characteristics of non-methane hydrocarbons and benzene series emission from common COF. Metayer et al. [18] studied COF and risk of lung cancer in women in rural Gansu, China. Tong et al. [19] explored the health risks of cooks inhaling COF with Chinese Sichuan cuisine as an example. These studies provide important evidence for the link between COF and health hazards. However, there are certain drawbacks, as they either focus on the composition and content of COF pollutants or only assess the health risks of specific regions or cuisines. Does the chemical composition and content of COF pollutants in different regions of China have similarities or differences, as well as differences in health risks? Thus far, few systematic studies have been conducted on the pollution characteristics and health risks of COF in different regions of China on the basis of the components and toxicology. The existence of these blind spots will make the personalized control of kitchen pollution in relevant areas lack of scientific basis, and it is difficult to develop targeted technologies or methods to degrade or eliminate pollutants.

**Figure 1:**
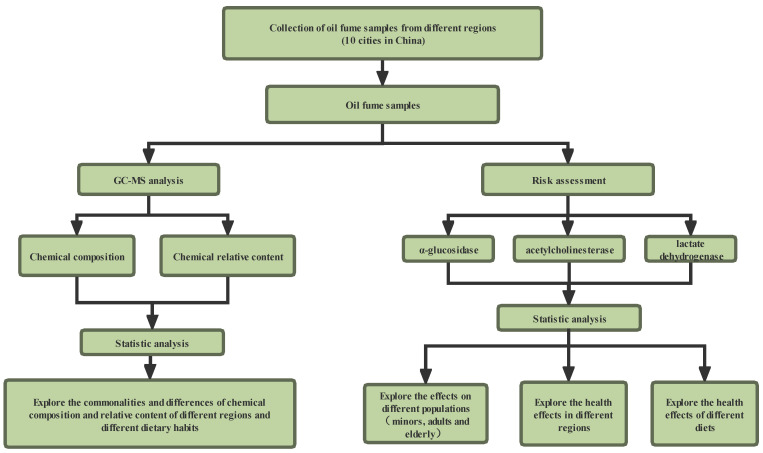
The design flowchart of this study.

Therefore, in this study, modern analytical methods such as GC–MS were used to analyze the types and contents of VOCs at the micro level in the condensates of COF from different regions. Then, in order to explore whether the types and contents of COF pollutants in different regions will cause different health risks, we detected the effects of COF condensates on the activities of lactate dehydrogenase (LDH), α-glucosidase, and acetylcholinesterase (AChE), which are closely related to health effects in human body, so as to clarify the toxicity characteristics of COF in different regions. The study design is shown in Figure 1. These studies may provide a scientific basis for solving the problem of kitchen environmental pollution. In fact, in addition to the kitchen environment, this study focused on many other scenarios such as offices, hospitals, shopping malls, and supermarkets with similar problems. That is, there is a lack of systematic regional comparison, and the commonalities and differences of different regions are not clear. The method or results of this study can provide a reference for the systematic and in-depth study of these scenarios.

## 2. Experiments

### 2.1. Sites and Sampling

**Figure 2:**
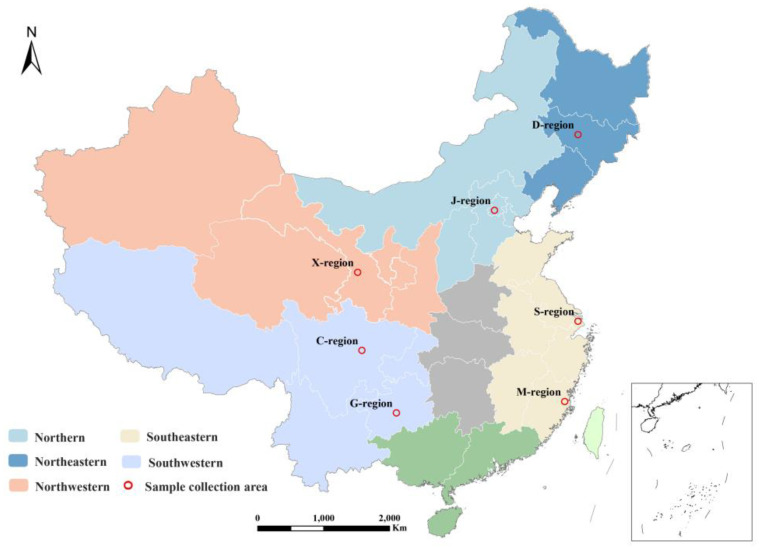
Distribution of sample collection sites. Note: Changchun represents Northeast China (D-region); Beijing and Tianjin represent North China (J-region); Lanzhou, Tongchuan and Qingyang represent Northwest China (X-region); Fuzhou and Shanghai represent Southeast China (M-region, S-region); Guiyang and Chengdu represent Southwest China (C-region, G-region).

From April to July 2021, we went to 10 typical representative cities of Changchun, Beijing, Tianjin, Lanzhou, Tongchuan, Qingyang, Guiyang, Chengdu, Fuzhou, and Shanghai to collect COF condensates in residential kitchens; the collection area distribution is shown in Figure 2.

In order to ensure the reliability of the research results, as well as to avoid cross-contamination of samples and exogenous interference as far as possible, the middle floor residents located in the internal buildings of the community were selected as the implementation objects (no other obvious pollution sources around the community). When sampling, the following households were excluded: (1) the randomly selected household registration was non-local; (2) households that did not use an exhaust system; (3) households that used an exhaust system but the amount of COF condensate was insufficient (25 mL). COF condensate was collected from families with similar cooking habits. Each of the 10 families were combined into one sample, and a total of 3 samples were collected from each region. Three samples were collected in each region. When collecting the samples, the basic information of the residence, including location, main cooking methods, taste characteristics, kitchen ventilation, and main cooking energy, were recorded (Table 1). Samples were collected in brown glass bottles, mixed well, sealed, and stored in a refrigerator at −80 °C until testing.

### 2.2. GC–MS Analysis

#### 2.2.1. Condition of Analysis

Each COF condensate sample was weighed accurately in a color-comparison tube of 0.1 g and then dissolved in 2 mL n-hexane for 5 min. After cooling, the filtrate through 0.20 μm filter membrane was taken for GC–MS analysis. GC–MS analysis was performed on an Agilent 7890A-5975C gas chromatography–mass spectrometer. Samples were separated using a DB5–MS (30 m × 0.25 mm × 0.25 μm) capillary column, and helium was selected as the carrier gas (purity ≥ 99.999%). The inlet temperature was set to 250 °C, and the interface temperature was set to 280 °C. The heating procedure was as follows: at the beginning, the temperature was kept at 80 °C for 3min, and then the temperature was raised to 250 °C at 20 °C per minute. After reaching 250 °C, the temperature was kept for 20 min. The ion source temperature was 250 °C, the four-stage rod temperature was 150 °C, the EI source voltage was 70 eV, the injection volume was 1 μL, the flow rate was 1 mL/min, and the scanning mode was full scanning.

#### 2.2.2. QA/QC

Blank experiment: Each batch of samples (not more than 10 samples) shall be a blank test; the determination results of internal standard concentration should not exceed the method detection limit. Otherwise, the reagent blank, instrument system, and pretreatment process should be checked.

Curve of calibration: The relative deviation of the relative response factor of the internal standard compound in the calibration curve should be less than or equal to 20%. Otherwise, indicating the injection port or column interference should be necessary maintenance. In continuous analysis, the intermediate concentration point of the calibration curve was analyzed every 24 h, and the relative standard deviation between the measured results and the actual concentration value should be less than or equal to 20%. Otherwise, the calibration curve must be redrawn.

Parallel sample: One pair of parallel samples should be analyzed for each batch of samples (up to 10 samples), and the relative deviation of the results of parallel samples should be less than 5%.

### 2.3. Toxicological Analysis

#### 2.3.1. Sample Preparation

A proper amount of each COF condensate was accurately weighed, and a solution of absolute ethanol and dimethyl sulfoxide (3:1) was used to make a series of sample solutions of 0.15, 0.17, 0.21, 13.23, 15.23, 18.59, 26.55, 30.51, and 37.19 μg/mL for subsequent toxicological evaluation. This series of concentrations were simulated for 1 day, 90 days, and 180 days exposure to COF for minors, adults, and the elderly, respectively. This series of concentrations was calculated by the following formulas:

C = M_i_/V (1)

M_i_ = C_0_ × f × T × t (2)

T = 8–10 mL/kg × G(3)

V = (G × 70%)/ρ(4)

where C represents daily concentration of COF in the lung after inhalation; M_i_ represents the amount of COF inhaled per person per day; V represents the volume of air that the lungs can take in or out; C_0_ represents the concentration of COF in kitchen air, in this study, the value was set as 5 mg/m^3^; f represents respiratory rate, the value was set as 15, 16, and 18 times/min for minors, adults, and elderly, respectively; T represents tidal volume; t represents average daily cooking time (150 min); G represents average body weight, the value was set as 57, 69.6, and 63.2 kg for minors, adults, and elderly, respectively; ρ represents human density (1.02 g/cm^3^).

#### 2.3.2. Determination of α-Glucosidase Activity

A modified version of the method followed by [20] was used to determine the inhibition rate of COF condensate on α-glucosidase. In particular, 50 μL of diluted sample, 25 μL of 3 U/mL α-glucosidase solution, and 25 μL of 1.5 mM PNPG were added to the 96-well plate in turn. After 30 min of reaction in the incubator at 37 °C, 100 μL of 200 mM Na_2_CO_3_ solution was added. After 10 min of reaction in the incubator at 37 °C, the samples were taken out and put into the optical absorption microplate reader to determine the absorbance at 405 nm. The activity of α-glucosidase was calculated using the following formula:

(5) $$\alpha \text{-}\mathrm{glucosidase}\;\mathrm{inhibition}\;\mathrm{rate}\;(\%)=(1-\frac{\;A_{\mathrm{sample}}-A_{\mathrm{sample}\;\mathrm{control}}}{A_{\mathrm{blank}}-A_{\mathrm{blank}\;\mathrm{control}}})\times 100$$

where $A_{\mathrm{sample}}$ represents the absorbance of sample and α-glucosidase, $A_{\mathrm{sample}\;\mathrm{control}}$ represents the absorbance of PBS with sample, $A_{\mathrm{blank}}$ represents the absorbance of PBS and α-glucosidase, and $A_{\mathrm{blank}\;\mathrm{control}}$ represents the absorbance of PBS.

#### 2.3.3. Determination of AchE Activity

The AchE inhibition rate of COF condensate was determined by referring to the literature method [21]. Phosphate buffer solution at 140 μL, sample at 20 μL, and 0.4 U/mL AchE at 15 μL were added to 96-well plates in turn. After 20 min inoculation at 4 °C, 7.5 mM ATCI 10 μL and 20 μL DTNB 20 μL were added. After 30 min inoculation at 37 °C, the absorbance was measured at 405 nm. The activity of AchE was calculated using the following formula:

(6) $$\mathrm{AChE}\;\mathrm{inhibition}\;\mathrm{rate}\;(\%)=(1-\frac{\;A_{\mathrm{sample}}-A_{\mathrm{sample}\;\mathrm{control}}}{A_{\mathrm{blank}}-A_{\mathrm{blank}\;\mathrm{control}}})\times 100$$

where $A_{\mathrm{sample}}$ represents the absorbance of sample and AChE, $A_{\mathrm{sample}\;\mathrm{control}}$ represents the absorbance of PBS with sample, $A_{\mathrm{blank}}$ represents the absorbance of PBS and AchE, and $A_{\mathrm{blank}\;\mathrm{control}}$ represents the absorbance of PBS.

#### 2.3.4. Determination of LDH Activity

LDH activity was tested according to the methods in the literature [22]. LDH solution (22.5 μg/L) and substrate (1.6 mM sodium pyruvate and 0.8 mM NADH mixed in equal volume) were prepared with phosphate buffer (pH 7.4). A total of 10 μL LDH solution and 10 μL sample were added to the 96-well plate in turn, and the mixture was incubated at 37 °C for 10 min. Then, 190 μL substrate was added, and it was oscillated evenly. After incubation at 37 °C for 10 min, the absorbance was immediately measured at 340 nm with a microplate reader. At the same time, the blank group, blank control group, and sample control group were set up. Each group was repeated three times, and the inhibition rate was calculated according to the following formula:

(7) $$\mathrm{LDH}\;\mathrm{inhibition}\;\mathrm{rate}\;(\%)=(1-\frac{\;A_{\mathrm{sample}}-A_{\mathrm{sample}\;\mathrm{control}}}{A_{\mathrm{blank}}-A_{\mathrm{blank}\;\mathrm{control}}})\times 100$$

where $A_{\mathrm{sample}}$ represents the absorbance of sample and LDH, $A_{\mathrm{sample}\;\mathrm{control}}$ represents the absorbance of PBS with sample, $A_{\mathrm{blank}}$ represents the absorbance of PBS and LDH, and $A_{\mathrm{blank}\;\mathrm{control}}$ represents the absorbance of PBS.

### 2.4. Statistical Analysis

Data analysis software developed by Agilent for GC–MS database retrieval was used to retrieve the name, molecular weight, structural formula, and relative content of VOCs in COF condensates in combination with the NIST 17.L mass spectrometry database. SIMCA 13.0 (Umetrics, Umea, Sweden) was used for multidimensional statistical analysis, including unsupervised principal component analysis (PCA), supervised least squares discriminant analysis (PLS-DA), and orthogonal partial least squares discriminant analysis (OPLS-DA). Excel 2021 was used for basic data analysis.

## 3. Results

### 3.1. Overall Analysis of VOCs in COF Condensates

In this study, the collected COF condensates were analyzed by a GC–MS method, and the corresponding total ion current (TIC) diagram was obtained. The name, molecular weight, structural formula, relative content, and other information of VOCs in the relevant COF condensate samples were obtained by TIC diagram and the NIST 17. L mass spectrometry database. Figure S1 shows the typical TIC diagrams of the samples in the seven regions. Each spectrum had good resolution and a different number of chromatographic peaks, indicating that the condensate of COF contained a large number of organic substances. The mass spectra of each peak in the TIC spectrum were compared with the standard mass spectra in NIST 17. L, and 174 VOCs were identified in 13 categories, which were classified as 15 saturated carboxylic acids (SCA), 10 saturated alkanes (SALK), 4 saturated alcohols (SALC), 8 saturated esters (SE), and 6 saturated aldehydes (SA) (Table S1); 29 unsaturated aldehydes (USA), 23 unsaturated carboxylic acids (USCA), 22 unsaturated esters (USE), 7 unsaturated alcohols (USALC), and 8 olefins (OF) (Table S2); and 17 heterocyclic compounds (HCC), 15 halogenated compounds (HGC), and 10 benzene series (BS) (Table S3).

**Figure 3:**
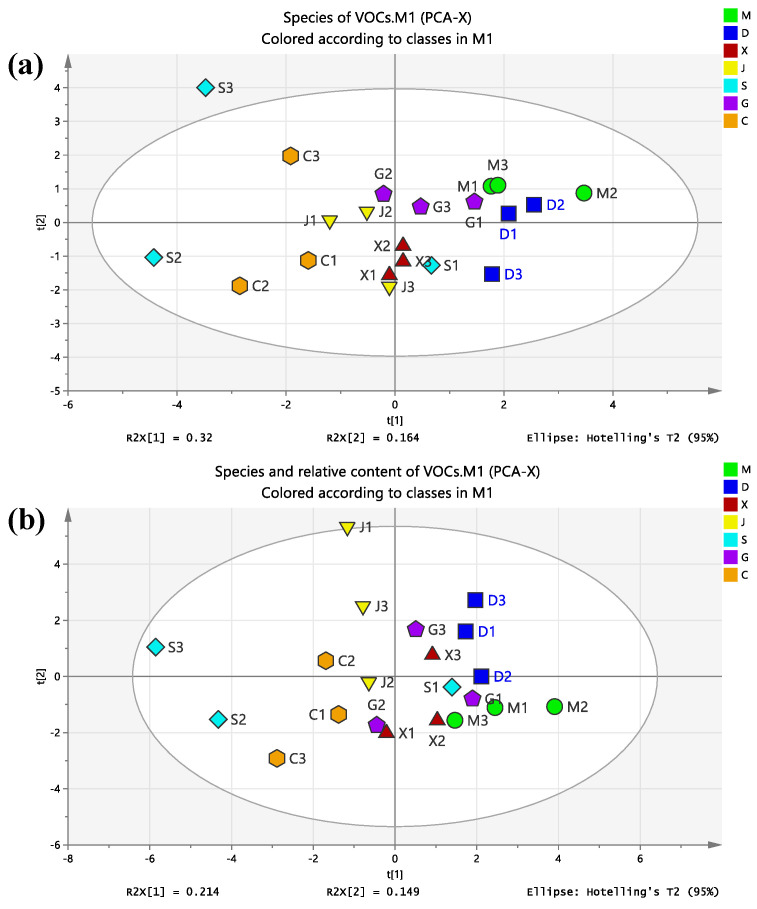
PCA-X diagram based on species (**a**) and ‘species + relative content’ (**b**) of VOCs from seven regions.

Further analysis showed that the same VOCs could be detected in the TIC spectra of different samples, but their peak intensities were not exactly the same. This shows that paying attention to the detection rate of each organic compound in different regions is not enough and that we should consider the difference of relative peak intensity. Therefore, in order to explore whether there are statistically significant differences in VOCs in COF condensate samples from different regions, this study used the PCA analysis module function of SIMCA software to conduct cluster analysis on the collected COF condensate samples on the basis of the 13 categories of organic compounds such as USA, USCA, and USE from the perspectives of ‘compound species’ (Figure 3a) and ‘compound species + relative content’ (Figure 3b).

It can be seen that when the COF in different regions were clustered on the basis of the types of VOCs, the samples from regions C, D, G, M, and X were clustered, and the boundaries between groups were obvious. Samples from other regions were dispersed (Figure 3a). When the COF in different regions were clustered on the basis of the types and relative contents of VOCs, the samples in regions C, D, J, M, and X were clustered into groups with obvious boundaries between groups. The data points of region G and region S were scattered (Figure 3b). The above results showed that although there were similar types of VOCs in the condensate samples of kitchen COF in the selected seven regions, there were differences in the number and relative content of compounds, and each region had its own unique compound composition.

### 3.2. Comparison of VOCs in the Seven Regions

In order to explore the similarities and differences of pollutants in different regions, the condensate samples were analyzed. The results showed that the number of organic compounds in each of the seven regions were in the order of S > C > X > J > G > D = M. The organic compounds detected were 71 in the S region, 68 in the C region, 60 in the X region, 49 in the J region, 48 in the G region, 31 in the D region, and 31 in the M region.

**Figure 4:**
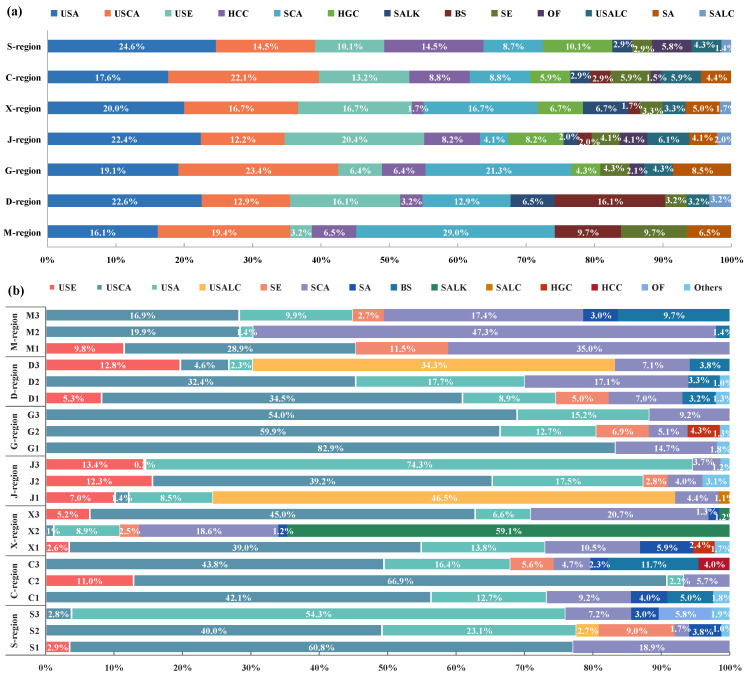
VOCs ^1^ in COF condensates of the 7 regions (**a**) species composition; (**b**) relative content. ^1^, Note: SCA, SALK, SALC, SE, SA, USA, USCA, USE, USALC, OF, HCC, HGC, and BS represent carboxylic acids, saturated alkanes, saturated alcohols, saturated esters, saturated aldehydes, unsaturated aldehydes, unsaturated carboxylic acids, unsaturated esters, unsaturated alcohols, olefins, heterocyclic compounds, halogenated compounds, and benzene series, respectively.

It can also be seen from Figure 4a that region J had the highest species composition, with 13 categories of VOCs detected. The species composition in region M was the least, and only eight compounds were detected. In the S region, X region, J region, and D region, USA accounted for the largest proportion of compounds; in regions C and G, the detection frequency of USCA compounds was the highest; M region had SCA containing the greatest number of compounds. USA, USCA, USE, HCC, and SCA appeared in seven regions of the COF condensate samples; these five categories of VOCs accounted for about 70% of all detected VOCs. Other compounds such as SALK, BS, SE, OF, USALC, and SA were detected in relatively small percentages. Furthermore, the relative content of compounds in the samples was analyzed (Figure 4b). The results showed that although there were similar kinds of organic pollutants in the samples from the seven regions, there were differences in the number of compounds and the relative content, and each region had its own unique compounds. SCA and USCA had relatively high contents in the M, D, X, G, and S regions; USA and USE had relatively high contents in region J; and the contents of USCA and USA were higher in the C region.

### 3.3. Comparative Analysis between the Southern and Northern Regions

Qinling Mountains and Huaihe River divide the mainland of China into two regions, the north and the south, and the two regions have huge regional differences in diet. In order to explore the differences and similarities of COF components in the north and south regions, the COF condensates in the north region (M + G + C + S) and the south region (D + X + J) were selected for PLS-DA and OPLS-DA cluster analysis based on VOCs species and relative content.

**Figure 5:**
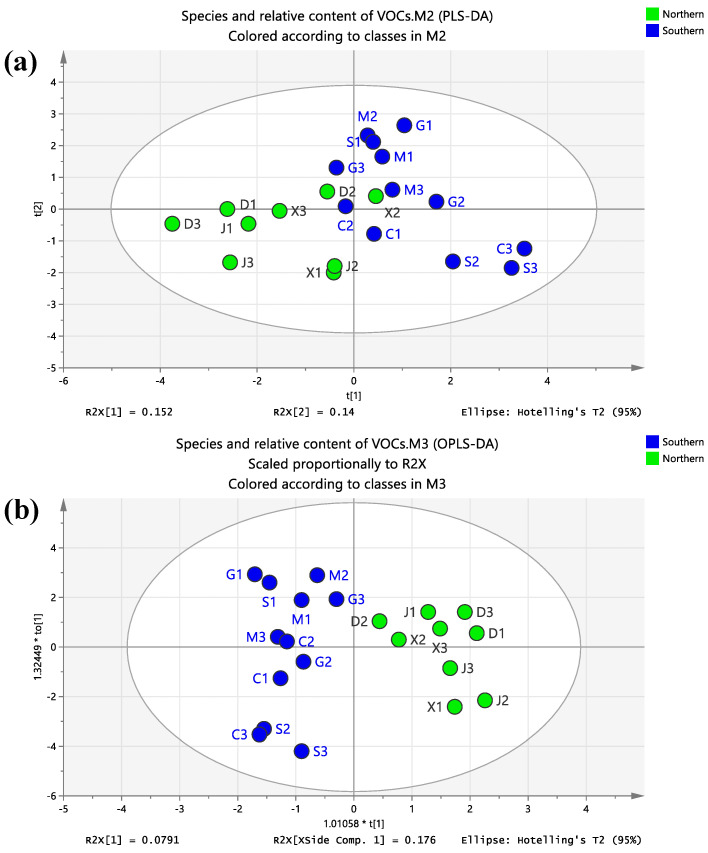
PCA-DA (**a**) and OPLS-DA (**b**) diagrams of northern and southern regions.

It can be seen from Figure 5 that the points in the southern region and the northern region were clustered into two categories, which were distributed on the left and right sides of the two score maps. The separation trend between the two groups in the OPLS-DA score map (Figure 5b) was more obvious than that in the PLS-DA score map (Figure 5a). The COF condensate sample points in the southern region were all distributed on the left side, and the sample points in the northern region were located on the right side of the score map. The above results showed that there were differences in the species and relative contents of COF pollutants in the south and north.

In terms of species (Table S4), there were 130 kinds of compounds in the south and 103 kinds of compounds in the north. There were 59 kinds of compounds in the two regions, including 17 kinds of USA; 12 kinds of USCA; 10 kinds of SCA; 6 kinds of USE; 3 kinds of SE; 2 kinds of SA, USALC, HGC, and HCC; and 1 kind of SALC, SALK, and BS. USA were the highest in both regions (26 in the South and 20 in the North). In addition, HCC (nine more in the south than in the north), USCA (eight more in the south than in the north), SCA (six more in the south than in the north), and USE (six more in the north than in the south) were more diverse (Table S5).

In terms of the relative content (Table S6), unsaturated carboxylic acid, saturated carboxylic acid, and unsaturated aldehyde had higher relative content in the two regions, while the relative content difference of saturated aldehyde, HGC, heterocyclic compound, and olefin was less and less than 6%. The relative contents of SALK and SALC were less than 2%. In addition, SE and SCA in the southern region were significantly higher than that in the northern region, and USE and USALC in the northern region were significantly higher than those in the southern region.

For the southern region (Tables S7 and S8), there were 89 and 86 kinds of compounds in the southeast (M-region, S-region) and southwest (C-region, G-region), respectively, and 45 kinds of compounds were the same, including 11 kinds of USCA, 10 kinds of USA, 7 kinds of SCA, 4 kinds of USE and HCC, 3 kinds of HGCs, 2 kinds of SA and USALC, and 1 kind of SE and BS. The COF in the two regions contained the most USA, USCA, and SCA. However, compared with the COF in the southwest region, the southeast region contained five more USA, four less USCA, and four more HCC, and there was little difference in the species of USA within three. In terms of relative content (Table S6), USCA were the highest in the two regions, but the most USCA in the southwest region were twice that in the southeast region, while the saturated carboxylic acid in the southeast region was significantly higher than that in the southwest region. In addition, there was little difference in the relative content of SA and USE between the two regions, and the relative content of SALK and SALC was very small and less than 1%. The relative content of halogenates and HCC in some samples in the southwest region was higher than that in the southeast region.

For the northern region (Table S9), there were 31, 60, and 49 volatile compounds in the COF condensates from the three regions of northeast (D-region), northwest (X-region), and north China (J-region), respectively. There were nine of the same compounds in the three regions, including USA, USCA, SE, USALC, and USE. The highest number of COF pollutants in the three regions were USA (7, 12, and 11 species, respectively), the middle number of species were USCA and USE, and the lowest number of species were SALC. However, there were 5 species of BS in Northeast China; only 1 species in Northwest and North China; and 4, 10, and 2 species of SCA for these three zones, respectively. There were no BS and HGC in Northeast China, and only OF in North China. In terms of the relative content, the USCA and USA of COF in the three regions were high, while the relative contents of saturated alkanes, HCC, OF, and SALC in their samples were low and less than 3%. In addition, the relative content of BS in Northeast China was significantly higher than that in the other two regions (Table S6), the saturated aldehyde in Northwest China was significantly higher than that in the other two regions, and the unsaturated ester in North China was significantly higher than that in the other two regions.

### 3.4. Comparative Analysis of Spicy Areas and Non-Spicy Areas

**Figure 6:**
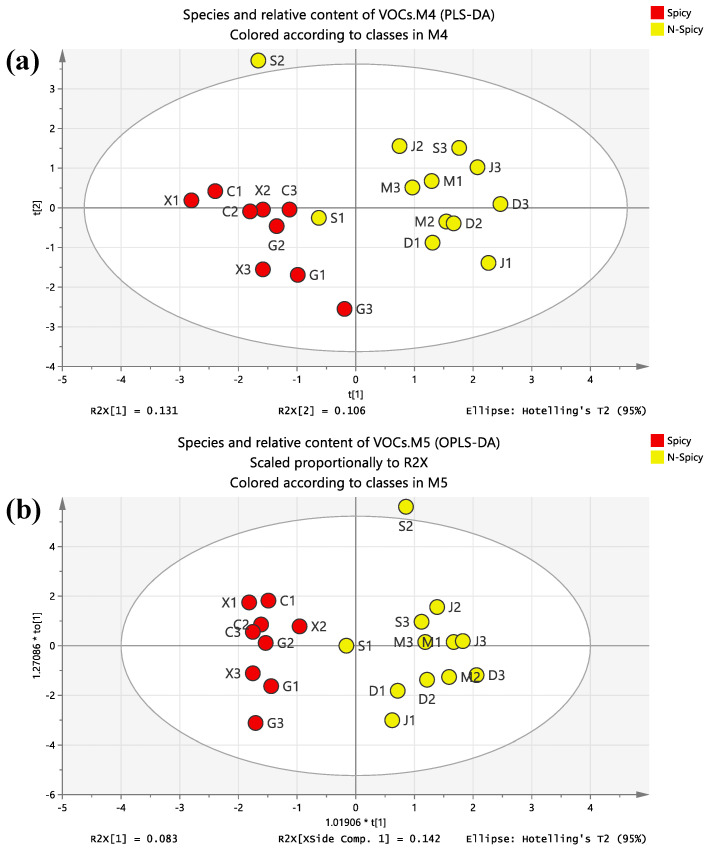
PLS-DA (**a**) and OPLS-DA (**b**) diagrams of spicy and non-spicy (N-Spicy) regions.

It can be seen from Figure 6 that the regions with and without spicy food had good separation. There were 111 and 108 kinds of organic compounds in spicy and non-spicy areas, respectively. Among them, there were 56 kinds of the same organic compounds, including 14 kinds of USA, 11 kinds of USCA, 9 kinds of SCA, 6 kinds of USE, 4 kinds of SA and HCC, 3 kinds of halogenates, 2 kinds of SE and USALC, and 1 kind of BS. The number of USA was the largest and was the same in spicy and non-spicy regions. There were 6 kinds of USCA, 10 kinds of SCA, 5 kinds of HCC, and 4 kinds of OF. There was little difference in the other types of compounds and less than three kinds.

In terms of the relative content, the higher contents of USCA, USA, and SCA were found in the COF from spicy areas compared with those from non-spicy areas, which was roughly the same as the species. However, the USCA of the COF from spicy areas were about twice as high as those from non-spicy areas, while the USALC, USE, and SE of the COF from non-spicy areas were significantly higher than those from non-spicy areas. In addition, the relative contents of SA, saturated alkanes, HGCs, HCC, OF, and SALC in the two regions were not much different, and the contents were relatively low.

### 3.5. Health Risk Assessment

#### 3.5.1. Analysis of Influence on α-Glucosidase Activity

α-Glucosidase has a wide range of sources and has important physiological functions in human glycogen degradation and carbohydrate metabolism of animals, plants, and microorganisms [23]. In the human body, α-glucosidase activity is directly related to blood glucose level, and excessive activation will accelerate the rise of blood glucose level, resulting in a significant increase in the risk of diabetes. On the contrary, excessive inhibition will lead to lower blood glucose levels, resulting in the emergence of hypoglycemia, thereby affecting human health [24]. In this study, in order to evaluate whether COF condensates in different regions have an impact on α-glucosidase activity, we simulated the exposure doses of COF condensates in different populations (minors, adults, and the elderly) and different exposure times (1 day, 3 months, and 6 months). On this basis, the in vitro test method was used to carry out the activity test of α-glucosidase.

**Figure 7:**
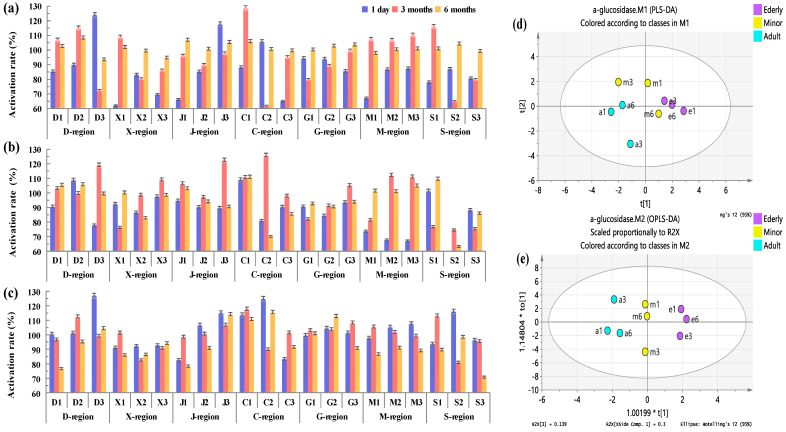
Inhibition rate of COF condensates on α-glucosidase for minors (**a**), adults (**b**), and the elderly (**c**), and their PLS-DA (**d**) and OP LS-DA (**e**) diagrams.

It can be seen from Figure 7a–c that the condensates of COF in different regions had significant inhibitory effects on α-glucosidase, and the inhibitory rates were between 61.73% and 129.25%, suggesting that the exposure of condensates of COF would produce potential hypoglycemia risks to human beings. Figure 7d,e shows the PLS-DA and OPLS-DA analysis diagram of α-glucosidase inhibitory activity. From the diagram, it is obvious that different groups were well separated, indicating that there were significant differences between groups, suggesting that there are significant differences in the impact of COF condensate exposure on different populations, thus implying that the risk of hypoglycemia in different populations after COF condensate exposure is different.

Briefly, in the minor group, 47.6% of the results showed that the inhibition of α-glucosidase activity by the amount of COF inhaled per capita for 6 months was greater than that of the other two inhalations; the data for adults and the elderly were 23.8% and 9.5%, respectively. The data of 57.1% in the adult group showed that the amount of COF inhaled for three months had the greatest inhibition of α-glucosidase activity, while the data of minors and the elderly had little difference, accounting for 38.1% and 42.9%, respectively. Compared with the data that the activity of α-glucosidase was inhibited by the daily intake of oil smoke per capita, the data in the elderly group accounted for 47.6%, followed by 38.1% in the adult group and 14.3% in the minors group. These results show that exposure to COF will inhibit the activity of α-glucosidase to a certain extent, resulting in a significant increase in the risk of hypoglycemia.

#### 3.5.2. Analysis of Influence on AchE Activity

**Figure 8:**
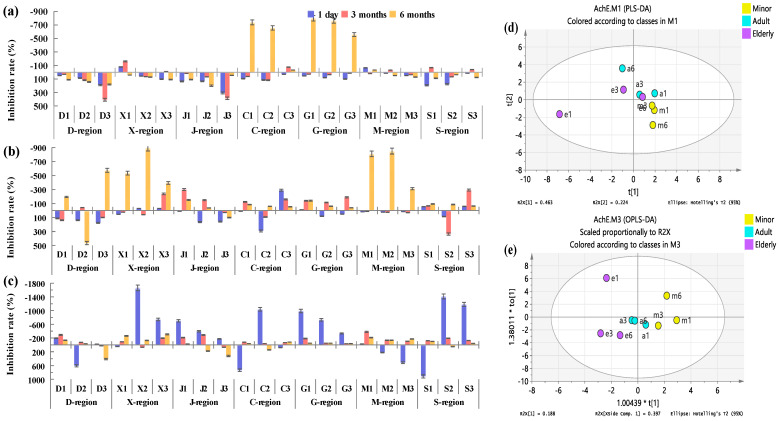
Inhibition rate of COF condensates on AchE of minors (**a**), adults (**b**), and the elderly (**c**) and their PLS-DA (**d**) and OP LS-DA (**e**) diagrams.

AchE is a key enzyme in biological nerve conduction that can degrade acetylcholine and ensure the normal transmission of nerve signals in vivo. The level of AchE is closely related to the cognitive function of the brain. The abnormal increase or decrease in its activity will indirectly affect the human cognitive function, thereby increasing the risk of dementia and other diseases [25]. In this study, in order to explore the effect of COF condensates from different regions of China on AchE activity, the activity test was carried out in vitro. The amount of COF inhaled by minors, adults, and the elderly per capita every day, per capita for 3 months, and per capita for 6 months was also selected as the experimental concentration dose (Figure 8). All kinds of sample points were gathered, and the aggregation effect of minor group was the best, followed by adult group, but in the elderly group, e1 was away from the other two points.

The daily intake of COF by minors, adults, and the elderly had both activation and inhibition effects on AchE. Among them, for minors and adults, AchE was mainly activated, and the data showed that the inhibition accounted for only 9.5% and 28.6%, respectively. For the elderly, the inhibition effect was mainly observed, and the data showing the activation effect accounted for 47.1%. The inhibition rate and activation rate were generally much larger than those of minors and adults (Figure 8a). The average amount of COF inhaled per capita for 3 months showed inhibition in minors, adults, and the elderly were 28.6%, 52.4%, and 85.7%, respectively. The inhibition rate of minors was essentially below 100%, and the inhibition rate of adults and the elderly was mainly between 100% and 300%. For the activation of 3 month inhalation of COF per capita, the activation rate was mostly below 100%, and for minors and adults, more than 300% at individual collection points (Figure 8b).

The amount of COF inhaled per capita for six months inhibited AchE activity in adults and the elderly, accounting for 90.5% and 76.2%, respectively. The inhibition rate of the elderly is concentrated between 10% and 200%. Although the inhibitory data of minors only accounted for 33.3%, the inhibitory rate generally exceeded 500% (Figure 8c). These results show that exposure to COF does affect the activity of acetylcholinesterase and thus has the risk of Alzheimer’s disease.

#### 3.5.3. Analysis of Influence on LDH Activity

**Figure 9:**
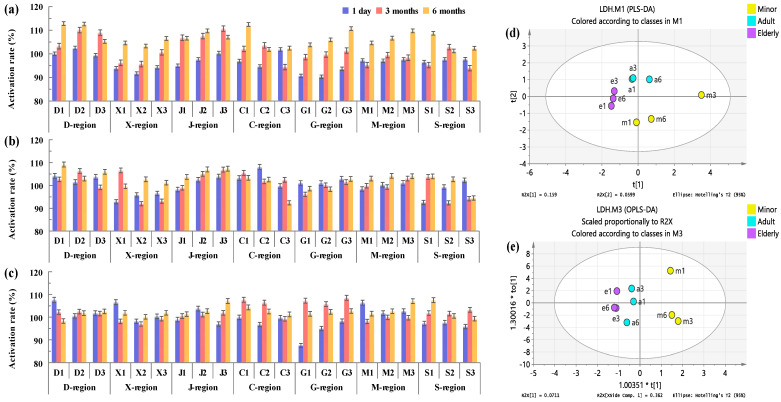
Inhibition rate of COF condensates on LDH of minors (**a**), adults (**b**), and the elderly (**c**), and their PLS-DA (**d**) and OP LS-DA (**e**) diagrams.

LDH, which widely exists in human tissues such as the heart, liver, kidney, skeletal muscle, and lungs, is an important oxidoreductase in the glycolysis pathway [26]. Abnormal changes in LDH activity in vivo usually indicate the occurrence of malignant tumors [27], epilepsy [28], and sepsis [29]. In this study, the effect of the collected COF condensate on the activity of LDH was measured in vitro, and the results were analyzed by PLS-DA and OPLS-DA models (Figure 9). Both models showed that the sample points of the three groups were clustered significantly, and the clustering effect of the elderly group was the best, followed by the adult group. The specific data of LDH activity are shown in Figure 9a–c. It can be seen that different COF condensate samples had different degrees of activation on LDH activity. In the minor group, the activation degree of LDH activity by the amount of COF inhaled per capita for 3 months and 6 months was significantly higher than that per capita per day. The results of the inhibition rate per capita for 6 months was higher than that of per capita per day and per capita for 3 months accounted for 76.2%. The inhibition rate per capita for 6 months was generally more than 100% or even more than 110% (Figure 9d).

In the adult group, there was little difference in inhibition rates among the three periods at most of the sampling sites. However, 52.4% of the results showed that the amount of COF inhaled by the average person in 6 months had a greater activation degree of LDH activity than the other two inhalations, and the data for 3 months and daily were 19% and 28.6%, respectively (Figure 9e).

In the elderly group, data from the sampling points showed that LDH activity was almost activated during the three time periods, with the highest activity at 6 months or 3 months per capita accounting for 42.9% and 38.1% of the total, respectively (Figure 9c). These results show that exposure to COF will activate LDH to a certain extent, resulting in a significant increase in the risk of tumors, epilepsy, and sepsis.

## 4. Discussion

### 4.1. Chemical Composition in COF and Its Impact on Health Risks

The chemical composition of COF has been one of the issues of wide concern. Chang et al. [30] found 220 compounds in COF, including 79 fatty acids, 13 alkanes, 16 OF, 38 aldehydes, 21 ketones, 15 alcohols, 27 esters, and 9 aromatic compounds. Compared with this study, since the detection object was COF condensate, although the total number of chemical components detected was 46 less, the detection rate of aromatic compounds was significantly higher. Lu et al. [15] found 11 kinds of VOC (alkanes, alcohols, aldehydes, USA, OF, oxides, ketones, nitrogen-containing compounds, furans, aromatics, and acids) in the respiratory region in the kitchen, including about 200 organic compounds. Compared with this study, 26 compounds of VOC species were found, but the types were similar. Schauer et al. [31] divided the collected COF into gas phase and particle phase. A total of 77 organic compounds were detected in the gas source, including 8 acids, 28 hydrocarbons, 20 aldehydes, 8 ketones, 9 aromatic hydrocarbons, and 4 other organic compounds. Hydrocarbons and aldehydes account for about 60% of organic compounds, while in this study, these two types of compounds accounted for only about 30%. A total of 49 organic compounds were detected in the collected particle phase source, including 19 acids, 15 hydrocarbons, 2 aldehydes, 2 ketones, 5 aromatic hydrocarbons, and 6 other organic compounds. Compared with the organic compounds detected in gas phase or particle phase, the types and species of VOCs in this study were relatively rich. Cheng et al. [32] obtained 55 kinds of VOCs, including 26 kinds of alkanes, 9 kinds of OF, and 16 kinds of aromatic hydrocarbons, which accounted for 92.7%. Cui [33] detected 68 kinds of VOCs, including 22 kinds of alkanes, 10 kinds of alkenes, 19 kinds of aromatic hydrocarbons, 2 kinds of alcohols, 7 kinds of aldehydes and ketones, 3 kinds of halogenated hydrocarbons, and 5 kinds of other compounds. Alkanes, alkenes, and aromatic hydrocarbons accounted for 75%. In the above two studies, hydrocarbons accounted for more than 70%, and in this study, these three kinds of alkanes, alkenes, and BS accounted for 16%, far less than the two studies. Li [34] selected the COF condensate for analysis and a total of 59 organic compounds were detected, including 14 acids, 12 hydrocarbons, 7 alcohols, 7 aldehydes, 3 ketones, 6 esters, 3 aromatic hydrocarbons, and 7 other organic compounds. In fact, the types of VOCs were roughly the same, but in our study, there were a greater number of VOCs. The reason is that the samples selected in this study were relatively large and contain multiple regions. In general, although a variety of components were detected in the COF condensate samples collected from seven regions of China, the types and quantities of VOCs were different from other studies.

The activity of α-glucosidase is directly related to blood glucose level [24]. Too high activity will increase the risk of diabetes, and too low will lead to hypoglycemia. AchE is closely related to the transmission of nerve signals [35]. Too much will lead to memory loss and slow response, causing Alzheimer’s disease [25]. Too little will cause acetylcholine accumulation in the body, leading to paralysis [36]. LDH is an important enzyme in the process of energy metabolism in the body [26], often used as an important index for diagnosis of visceral diseases [37]. It is also closely related to tumor metabolism, growth, proliferation, invasion, and metastasis [26]. Skeletal muscle injury and leukemia are also associated with it [38,39]. It is a matter of concern as to how different chemical compositions can affect health effects, as well as how different chemical compositions can affect health effects. Therefore, in this study, we used regions (southern region vs. northern region, spicy region vs. non-spicy region) as grouping variables for PLS-DA and OPLS-DA analysis. It can be clearly seen from Figures S2–S4 that different groups were well separated, and there were significant differences between groups, indicating that there were significant differences in the effects of COF condensate exposure on α-glucosidase, AchE, and LDH activities in different regions.

Data comparison showed that the α-glucosidase inhibition rate of COF in the north was higher than that in the south, which suggested that the risk of hypoglycemia was higher in the north than in the south. The results of the α-glucosidase inhibition rate in adults and the elderly were opposite, suggesting that the risk of hypoglycemia in men was higher than that in northern China. The average inhibition rate of α-glucosidase in minors and the elderly by inhaling COF in non-spicy areas was higher than that in spicy areas, and that in adults was higher in spicy areas than in non-spicy areas. These results suggest that different populations in spicy and non-spicy regions have different sensitivities to α-glucosidase, leading to different hypoglycemic risks.

Comparing the proportion of data on the activation of AchE by inhalation of COF condensate in three groups of people, it was found that the proportion of data showing activation in the southern region was almost completely greater than the corresponding value in the northern region. This proportion of spicy areas was also greater than that of non-spicy areas. These results suggested that exposure to COF in southern and spicy areas was associated with a higher risk of neurological diseases such as Alzheimer ‘s disease than in northern and non-spicy areas.

In the cases of single-day, 3-month, and 6-month exposure, the inhibition rate of LDH in the northern region was greater than that in the southern region for minors. The trend of adults and the elderly was the same, that is, at three months per capita and six months per capita, the north was larger than the south. For minors and adults, the average inhibition rate of the amount of smoke inhalation on LDH in the non-spicy areas was higher than that in the spicy areas. For the elderly, the non-spicy areas were greater than the spicy areas in terms of per capita single-day and three-month exposure. However, at 6 months of exposure, it was larger in spicy areas than in non-spicy areas. These results showed that different regions, populations, and exposure had different effects on LDH activity and ultimately led to different health risks.

### 4.2. Influencing Factors of COF Components

China has a vast territory. Due to differences in climate, products, and customs, cooking methods vary from place to place, and each method has its own special features. In this study, fourteen main cooking styles were involved in the seven regions, among which stir-frying, blasting, burning, stewing, and steaming are the existing cooking methods in both southern and northern regions. Blanching, soy, shao, fried, and sauce are the unique techniques in the north, while stir-frying, boiling, and mixing are the unique styles in the south. Our study found that there were differences in the number and relative content of compounds in COF pollutants between different regions, especially in the north and south, and cooking habits may be an important reason for this result. Ho et al. [40] collected exhaust gas samples of different cooking styles from the kitchen. The data showed that the acrolein content of Sichuan spicy restaurants was the highest; the content of nonanal, heptanal, and acrolein in Korean barbecue kitchens was higher; the content of nonanal and acetaldehyde in western fast food chains was higher; and the acrolein content of western steak restaurants was the highest. Svendsen et al. [41] assessed the exposure of COF in different kitchens in Norway. The local restaurants had the highest levels of aldehydes, while the burger chains had the lowest levels of aldehydes. Studies by Peng et al. [42] showed that changing cooking methods can significantly alter aldehyde emissions, with frying having the highest levels of total aldehyde.

In China, in addition to cooking habits, eating habits are also varied. Cold regions are mainly salty, and people like to eat onion, ginger, and garlic. People in rainy and humid areas such as Guizhou and Sichuan like spicy food, whereas people in Guangdong and other places like to eat a light diet. Our study found that there were significant differences in the composition of kitchen COF fumes between spicy and non-spicy areas. Wang et al. [43] evaluated the composition characteristics of cooking emissions under different combinations of cooking oils, seasonings, and dishes. Benzene was produced in soybean oil, peanut oil, and rapeseed oil, but the content was different. For soybean oil and peanut oil, the contents of acetaldehyde, propionic aldehyde, butyraldehyde, and pentanal increased after the addition of chili powder compared with the heating of oil. The VOC emissions from chili pork were much higher than those from eggs and tomatoes. The release of benzene, formaldehyde, acrolein, isoprene, and acetonitrile was much higher in chili pork than in egg and tomato dishes. Peng et al. [42] observed the concentration composition of 13 aldehydes in oil fumes by changing the types of edible oils (soybean oil, sunflower oil, rapeseed oil, and palm oil), finding that there were differences in the concentration composition of total aldehydes and single aldehydes in different edible oils. It was also found that the acetaldehydes; hexanals; and trans, trans-2, 4-decadienal produced by cooking pork tenderloin were higher than those produced by cooking potatoes. The above research shows that the types of edible oil and ingredients are important factors affecting the composition of volatile components of cooking fumes, and the types of edible oil and ingredients selected in daily cooking are related to dietary habits, further indicating that dietary habits are one of the main factors affecting the composition of cooking fumes.

While collecting samples, we recorded the ventilation habits of residents. Some residents used to only turn on the range hood when cooking, and some users used to open the window while turning on the range hood. For these two habits, we did not find significant differences in the types and relative contents of organic pollutants in COF condensates. The households we surveyed used natural gas or electricity as cooking energy, and the results did not find significant differences in the composition of organic pollutants in the COF condensate. However, studies have shown that different cooking energy sources can result in different VOC composition in cooking fumes [44,45].

### 4.3. Study Limitation

This study collected COF condensate from 10 cities in different regions of China to carry out related research. However, the actual sampling process is full of difficulties, and sample collection is not easy. Therefore, the sampling principle is as follows: 10 households with similar cooking habits were taken as one sample, and 3 samples were collected from each city. Although this sampling method can reflect the actual situation of the sample as a whole, it also has some drawbacks, such as introducing major bias, and the number of samples was small. These drawbacks may affect the results of the analysis. In the follow-up research work, we can find ways to increase the number of samples to make the results more representative.

## 5. Conclusions

In this study, by analyzing the COF condensates in 10 representative cities in 7 regions, 174 kinds of VOCs were identified. It was found that the types of organic compounds in the seven regions were S > C > X > J > G > = > M, which were 71, 68, 60, 49, 48, 31, and 31, respectively. The relative contents of SCA and USCA were higher in the M, D, X, G, and S regions; USA and USE were higher in the J region; and USCA and USA were higher in the C region. There were differences in the types and relative contents of VOCS in southern and northern COF pollutants. In terms of species, there were 130 compounds in the southern region, 103 compounds in the northern region, and 59 compounds commonly found in both regions. In terms of relative content, SE and SCA in the southern region were significantly higher than those in the northern region, and USE and USALC in the northern region were significantly higher than those in the southern region. There were also differences in the types and relative contents of VOCs in COF between spicy and non-spicy areas. In terms of species, spicy and non-spicy areas contained 111 and 108 organic compounds, respectively, of which 56 were common. From the relative content, the USCA in the spicy area was about two times higher than that in the non-spicy area, while the USALC, USE, and SE were significantly higher in the non-spicy areas than in the spicy areas. Health risk assessment showed that COF could inhibit α-glucosidase, activate LDH, and exert both inhibitory and activating effects on AchE. This suggests that exposure to COF has risks of hypoglycemia, Alzheimer’s disease, tumors, epilepsy, and sepsis. Moreover, the inhibition rate or activation rate of COF in different regions was significantly different, indicating that the risk of related diseases was different.

## Figures and Tables

**Table 1:** Basic information for different sampling sites.

Collection Sites	Representative City	Taste Characteristics	Main Cooking Methods	Ventilation Equipment	Cooking Energy
Northeast China (D-region)	Changchun (D1)	Salty, sweet, and strong taste.	Boil, stew, stew, and grill	Windows and range hoods	Natural gas
	Changchun (D2)		Stew, boil, sauce, and stir	Windows and range hoods	Natural gas
	Changchun (D3)		Burn, stew, boil, and sauce	Windows and range hoods	Natural gas
North China (J-region)	Tianjin (J1)	Salty, fresh, light, sweet, and sour.	Explosion, fry, and burn	Windows and range hoods	Natural gas
	Tianjin (J2)		Blast, fry, burn, and grill	Windows and range hoods	Natural gas
	Beijing (J3)		Explosion, fry, and burn	Range hoods	Natural gas
Northwest China (X-region)	Lanzhou (X1)	The flavor is distinct, and the aroma is outstanding.	Fast, lightly steamed, and fried	Windows and range hoods	Natural gas
	Tongchuan (X2)		Fried, fried, steamed, and fried	Windows and range hoods	Natural gas
	Qingyang (X3)		Steam, fry, stew, and warm mixing	Windows and range hoods	Electricity
Southwest China (G-region)	Guiyang (G1)	Spicy alcohol, acid fresh, and strong taste.	Fried, boiled, stewed, and mixed	Windows and range hoods	Natural gas
	Guiyang (G2)		Fried, boiled, stewed, and mixed	Windows and range hoods	Electricity
	Guiyang (G3)		Fried, boiled, stewed, and mixed	Windows and range hoods	Natural gas
Southwest China (C-region)	Chengdu (C1)	Fresh, mellow, and spicy.	Fried, exploded, and cooked	Windows and range hoods	Natural gas
	Chengdu (C2)		Fry, explode, stir, and explode	Windows and range hoods	Natural gas
	Chengdu (C3)		Fry, slide, boil, and stir	Range hoods	Natural gas
Southeast China (M-region)	Fuzhou (M1)	Fresh and alcohol; no greasy meat ointment.	Steam, fry, and stew	Windows and range hoods	Natural gas
	Fuzhou (M2)		Steam, fry, boil, and soak	Windows and range hoods	Natural gas
	Fuzhou (M3)		Steam, sauté, and fry	Windows and range hoods	Natural gas
Southeast China (S-region)	Shanghai (S1)	Thick oil red sauce; moderate taste.	Sauté, braise, fry, and boil	Range hoods	Natural gas
	Shanghai (S2)		Sauté, braise, fry, and boil	Windows and range hoods	Natural gas
	Shanghai (S3)		Sauté, braise, fry, and boil	Range hoods	Natural gas

## Data Availability

Data are contained within this article.

## Supplementary Materials

The following are available online at https://www.mdpi.com/article/10.3390/foods12010106/s1, Figure S1: TIC spectra of the representing samples from seven regions. Figure S2: Statistical analysis of the influence of COF condensates in different regions on α-glucosidase activity. Figure S3: Statistical analysis of the influence of COF condensates in different regions on AchE activity. Figure S4: Statistical analysis of the influence of COF condensates in different regions on LDH activity. Table S1: Saturated VOCs detected from oil fume condensates in seven regions. Table S2: Unsaturated VOCs detected from oil fume condensates in seven regions. Table S3: Heterocyclic compounds, halides, and benzene series detected from oil fume condensates in seven regions. Table S4: The same compounds in the northern and southern regions. Table S5: Different compounds in the northern and southern regions. Table S6: Relative content of compounds in the northern and southern regions. Table S7: The same compounds in the southeast (M-region + S-region) and southwest (C-region + G-region) regions. Table S8: The different compounds in the southeast (M-region+S-region) and southwest (C-region + G-region) regions. Table S9: Compounds in the northern region.
