# Supplementary material for: The Chemical Composition Characteristics and Health Risk Assessment of Cooking Fume Condensates from Residential Kitchens in Different Regions of China
Source: Foods. 2022 Dec 25;12(1):106. doi: 10.3390/foods12010106 (PMC9818279; doi:10.3390/foods12010106)
Supplement: Supplementary file 1 [file foods-12-00106-s001.zip › foods-2063527-supplementary-revised (1).pdf]

Support materials

The chemical composition characteristics and health risk assessment of cooking fume condensates from residential kitchens in different regions of China

Qinghong Liu[1, #], Xiaofang Zhang[1, #], Yan yang[2], Qiuxia Tan[2], Liting Zheng[2], Hongwei Lou[1], Huaguo Chen[2,*], Qin Yang[1,*]

[1]College of Civil Engineering, Guizhou University, Guiyang, 550025, P.R. China

[2.] Guizhou Engineering Laboratory for Quality Control & Evaluation Technology of Medicine, Guizhou Normal University, Guiyang 550001, China

[#] These authors are contributed equally to this work.

[*] Corresponding author at: College of Civil Engineering, Guizhou University, Guiyang, 550025, P.R. China.

E-mail addresses for Qin Yang: qyang7@gzu.edu.cn

E-mail addresses for Huaguo Chen: chenhuaguo@gznu.edu.cn

Contents list

Support materials 1
Figure S1. TIC spectra of the representing samples from seven regions. 3
Figure S2. Statistical analysis of the influence of COF condensates in different regions on α−glucosidase activity. 4
Figure S3. Statistical analysis of the influence of COF condensates in different regions on AchE activity 5
Figure S4. Statistical analysis of the influence of COF condensates in different regions on LDH activity 6
Table S1 Saturated VOCs detected from oil fume condensates in seven regions. 7
Table S2 Unsaturated VOCs detected from oil fume condensates in seven regions. 8
Table S3 Heterocyclic compounds, halides, and benzene series detected from oil fume condensates in seven regions 10
Table S4 Same compounds in the Northern and Southern. 12
Table S5 Different compounds in Northern (N) and Southern (S). 14
Table S6 Relative Content of Compounds in Northern and Southern Regions 17
Table S7 The same compounds in southeast (M-region+S-region) and southwest (C-region + G-region). 18
Table S8 The different compounds in southeast (M-region+S-region) and southwest (C-region + G-region). 20
Table S9 Compounds in Northern. 23

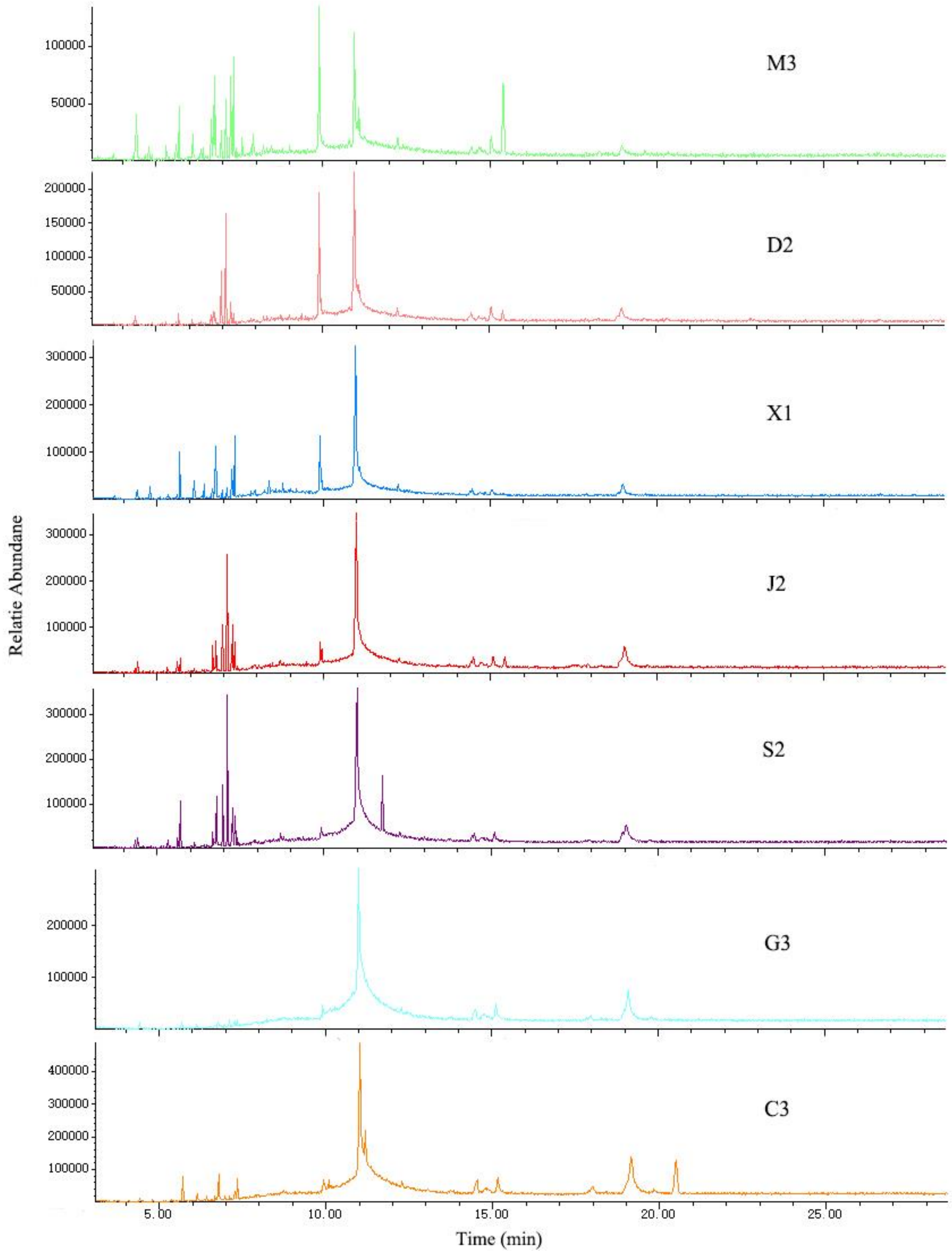

Figure S1. TIC spectra of the representing samples from seven regions. The sampling site is M-region, representative city is Fuzhou (M3); the sampling site is D-region, representative city is Changchun (D2); the sampling site is X-region, representative city is Lanzhou (X1); the sampling site is J-region, representative city is Tianjin (J2); the sampling site is S-region, representative city is Shanghai (S2); the sampling site is G-region, representative city is Guiyang (G3); the sampling site is C-region, representative city is Chengdu (C3).

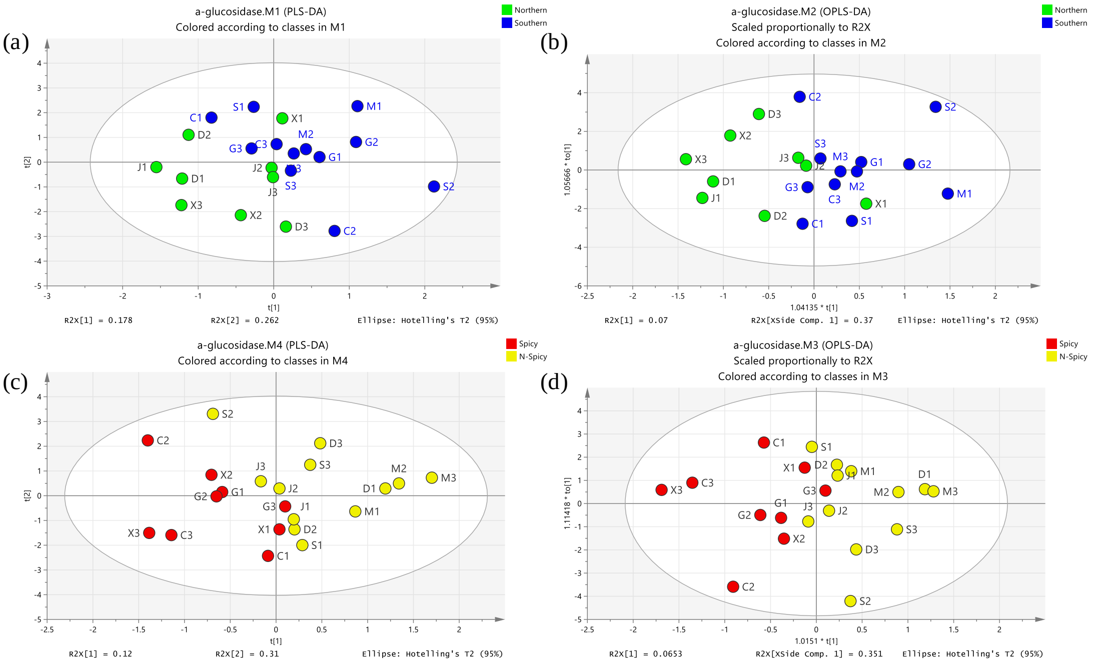

Figure S2. Statistical analysis of the influence of COF condensates in different regions on α-glucosidase activity. PLS-DA analysis of northern and southern (a), OPLS-DA analysis of northern and southern (b), PLS-DA analysis of spicy regions and non-spicy regions (c) and OPLS-DA analysis of spicy regions and non-spicy regions (d).

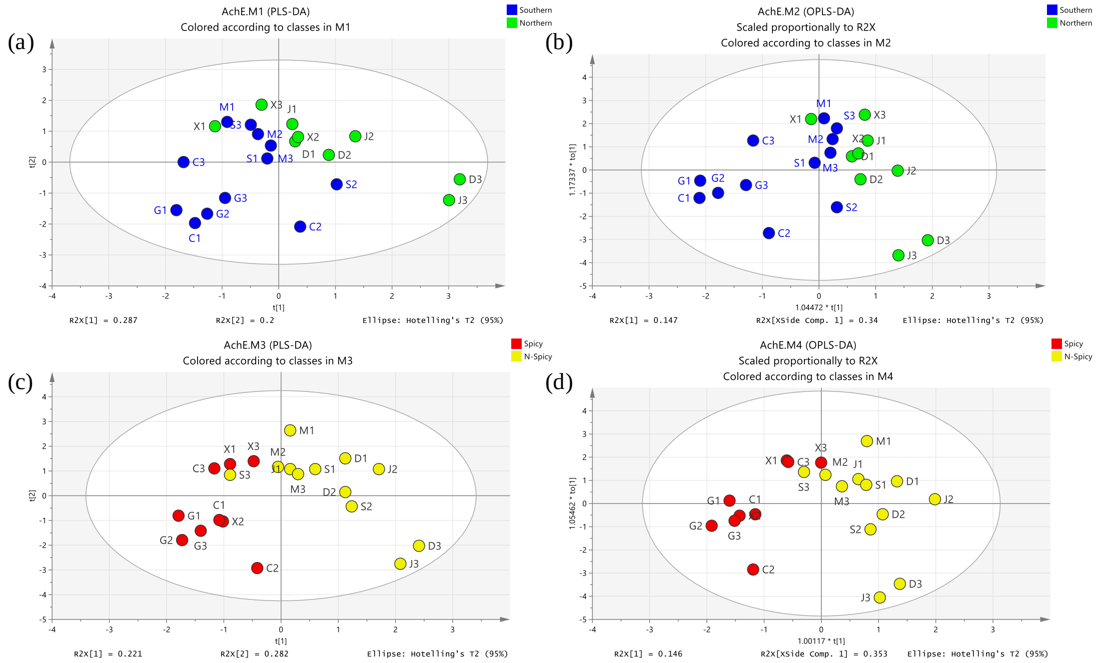

Figure S3. Statistical analysis of the influence of COF condensates in different regions on AchE activity. PLS-DA analysis of northern and southern (a), OPLS-DA analysis of northern and southern (b), PLS-DA analysis of spicy regions and non-spicy regions (c) and OPLS-DA analysis of spicy regions and non-spicy regions (d).

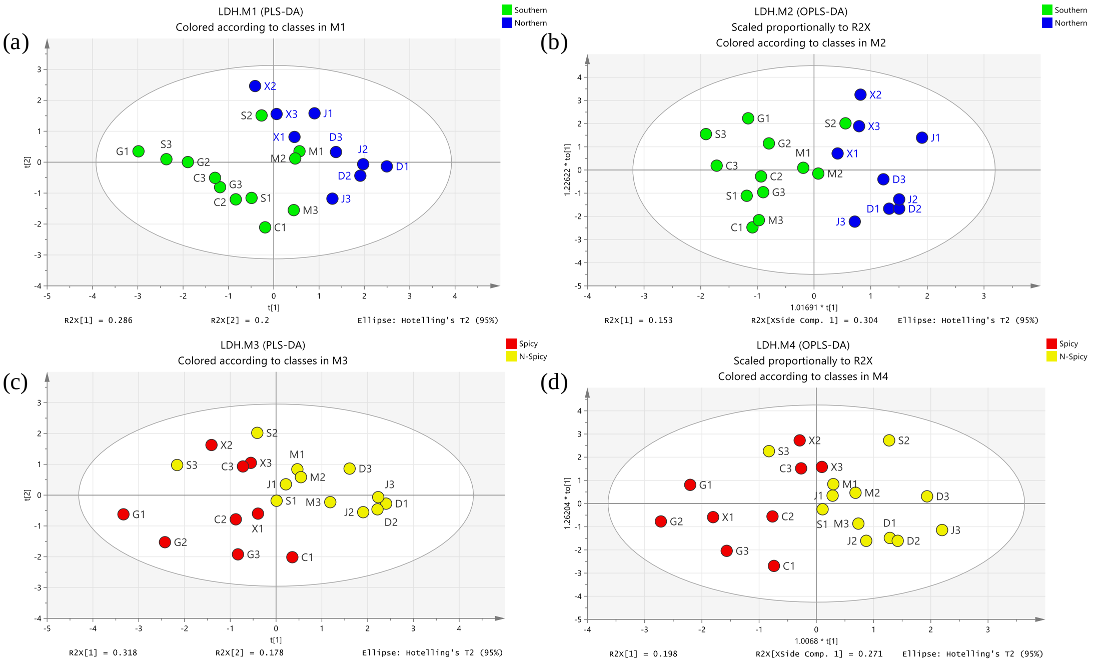

Figure S4. Statistical analysis of the influence of COF condensates in different regions on LDH activity. PLS-DA analysis of northern and southern (a), OPLS-DA analysis of northern and southern (b), PLS-DA analysis of spicy regions and non-spicy regions (c) and OPLS-DA analysis of spicy regions and non-spicy regions (d).

Table S1 Saturated VOCs detected from oil fume condensates in seven regions.

SCA	SALK	SE
Tridecanoic acid	Oxirane, hexyl-	Tributyl acetylcitrate
Tetradecanoic acid	Oxirane, 2-butyl-3-methyl-, cis-	Oxiraneundecanoic acid, 3-pentyl-, methyl ester, trans-
Pentanoic acid, 3-methyl-	Hexacosyl nonyl ether	Octadecanoic acid, 2,3-dihydroxypropyl ester
Pentadecanoic acid	Docosane, 7-butyl-	Hexadecanoic acid, ethyl ester
Octanoic acid	Cycloeicosane	Hexadecanoic acid, 2-hydroxy-1-(hydroxymethyl)ethyl ester
Octadecanoic acid	Cyclodecane	Heptadecanoic acid, heptadecyl ester
Nonanoic acid	Bicyclo[3.1.1]heptane, 2,6,6-trimethyl-, [1R-(1.alpha.,2.alpha.,5.alpha.)]-	Glycidyl palmitate
n-Hexadecanoic acid		Butyric acid, 2-pentadecyl ester
Hexanoic acid	13-Oxabicyclo[10.1.0]tridecane	**SALC**
Heptanoic acid	1,2-15,16-Diepoxyhexadecane	Decanal
Heptadecanoic acid	**SA**	Cyclohexanol, 3-methyl-
Dodecanal	Octanal	1,15-Pentadecanediol
Azelaic acid	Nonanal	Ethanol, 2-(2-butoxyethoxy)-
15-Hydroxypentadecanoic acid	D-erythro-Pentose, 2-deoxy-	Behenic alcohol
12-Hydroxydodecanoic acid	Octadecanal	

Table S2 Unsaturated VOCs detected from oil fume condensates in seven regions.

USA	USCA	USE
Oxalic acid, allyl dodecyl ester	Z-8-Methyl-9-tetradecenoic acid	Z-(13,14-Epoxy)tetradec-11-en-1-ol acetate
cis-9-Hexadecenal	Z-7-Tetradecenoic acid	Undec-10-ynoic acid, undecyl ester
cis-11-Hexadecenal	Undecylenic acid	Undec-10-ynoic acid, tetradecyl ester
cis,cis-7,10,-Hexadecadienal	trans-Traumatic acid	Undec-10-ynoic acid, dodecyl ester
9-Tetradecenal, (Z)-	trans-13-Octadecenoic acid	trans-9-Octadecenoic acid, pentyl ester
9-Octadecenal, (Z)-	Palmitoleic acid	Pentanoic acid, 10-undecenyl ester
9-Octadecenal	Oleic Acid	Oxalic acid, cyclobutyl hexadecyl ester
9,17-Octadecadienal, (Z)-	Linoelaidic acid	n-Propyl 11-octadecenoate
9,12-Octadecadienal	E-9-Tetradecenoic acid	Methyl 7,8-octadecadienoate
9(E),11(E)-Conjugated linoleic acid	E,E-10,12-Hexadecadien-1-ol acetate	Glycidyl palmitoleate
8-Hexadecenal, 14-methyl-, (Z)-	Cyclopentaneundecanoic acid	Glycidyl (Z)-9-Heptadecenoate
7-Hexadecenal, (Z)-	cis-Vaccenic acid	Glycerol 1-palmitate
2-Undecenal, E-	cis-7-Hexadecenoic acid	Glutaric acid, tridec-2-yn-1-yl dec-4-enyl ester
2-Undecenal	cis-13-Octadecenoic acid	Decanoic acid, silver(1+) salt
2-Tridecenal, (E)-	9-Oxononanoic acid	Carbonic acid, decyl vinyl ester
2-Octenal, (E)-	9-Octadecenoic acid	9-Octadecenoic acid (Z)-, methyl ester
2-Nonenal, (E)-	9-Hexadecenoic acid	9-Octadecenoic acid (Z)-, 2-hydroxyethyl ester
2-Heptenal, (Z)-	9-Eicosenoic acid, (Z)-	9-Octadecenoic acid (Z)-, 2-hydroxy-1-(hydroxymethyl)ethyl ester
2-Dodecenal, (E)-	9,12-Octadecadienoic acid (Z,Z)-	9-Octadecenoic acid (Z)-, 2,3-dihydroxypropyl ester
2-Dodecenal	2-Octenoic acid	9,12-Octadecadienoic acid (Z,Z)-, 2-hydroxy-1-(hydroxymethyl)ethyl ester
2-Decenal, (Z)-	2-Nonynoic acid	1-cis-Vaccenoylglycerol
2-Decenal, (E)-	2-Dodecenoic acid	1,1-Dodecanediol, diacetate
2,4-Nonadienal, (E,E)-	17-Octadecynoic acid	USALC
2,4-Dodecadienal, (E,E)-	OF	Z,Z-3,13-Octadecadien-1-ol
2,4-Decadienal, (E,Z)-	E,Z-1,3,12-Nonadecatriene	Z,E-3,13-Octadecadien-1-ol
2,4-Decadienal, (E,E)-	Cyclodecene	E-2-Octadecadecen-1-ol
2,4-Decadienal	8-Heptadecene	9,12-Octadecadien-1-ol, (Z,Z)-

13-Tetradecenal	7-Tetradecenal, (Z)-	6,9,12-Octadecatrien-1-ol
13-Octadecenal, (Z)-		2-Methyl-Z,Z-3,13-octadecadienol
		12-Methyl-E,E-2,13-octadecadien-1-ol

Table S3 Heterocyclic compounds, halides, and benzene series detected from oil fume condensates in seven regions.

HCC	HGC	BS
Stevioside	Trichloroacetic acid, undec-2-enyl ester	Phthalic acid, isobutyl octadecyl ester
Oxalic acid, cyclohexylmethyl octyl ester	Trichloroacetic acid, undec-10-enyl ester	Phthalic acid, ethyl tetradecyl ester
Oxacyclohexadecan-2-one	Oleyl alcohol, trifluoroacetate	Phthalic acid, butyl undecyl ester
Furan, 2-pentyl-	Oleyl alcohol, chlorodifluoroacetate	Phthalic acid, 5-methylhex-2-yl heptadecyl ester
D-chiro-Inositol,*	Octanoic acid, silver(1+) salt	Estra-1,3,5(10)-trien-17.beta.-ol
Cyclododecanone, 2-methylene-	Carbonic acid, 2,2,2-trichloroethyl undec-10-enyl ester	Bis(2-ethylhexyl) phthalate
cis-Dihydrocarvone	Acetic acid, trifluoro-, dodecyl ester	1,4-Benzenedicarboxylic acid, bis(2-ethylhexyl) ester
9-Oxabicyclo[6.1.0]nonane, cis-	9,12-Octadecadienoyl chloride, (Z,Z)-	1,3-Benzenedicarboxylic acid, bis(2-ethylhexyl) ester
9-Methylbicyclo[3.3.1]nonane	7-Heptadecene, 1-chloro-	1,2-Benzenedicarboxylic acid, mononoyl ester
9-Borabicyclo[3.3.1]nonane, 9-hydroxy-	6-Methyl-2-heptanol, trifluoroacetate	
8-Oxabicyclo[5.1.0]octane	4-Chloro-3-n-hexyltetrahydropyran	
4-Cyclononen-1-one	2- Chloropropionic acid, hexadecyl ester	
2H-Pyran, 3,4-dihydro-	(Z)-Tetradec-11-en-1-yl 2,2,2-trifluoroacetate	
2,5,6-Trimethyl-1,3-oxathiane	(E)-Tetradec-11-en-1-yl 2,2,3,3,4,4,4-heptafluorobutanoate	
2(1H)-Naphthalenone, octahydro-, trans-	(E)-tetradec-11-en-1-yl 2,2,3,3,3-pentafluoropopanoate	

1,6-Cyclodecadiene		
.alpha.-D-Mannopyranoside, methyl 3,6-anhydro-		

Table S4 Same compounds in the Northern and Southern.

Spcies（numbers）	Chemical	Molecular weight	Chemical formula
SALC	Decanal	156	$C_{10}H_{20}O$
SA	Octanal	128	$C_8H_{16}O$
SA	Nonanal	142	$C_9H_{18}O$
SCA	Hexanoic acid	116	$C_6H_{12}O_2$
SCA	Octanoic acid	144	$C_8H_{16}O_2$
SCA	Nonanoic acid	158	$C_9H_{18}O_2$
SCA	n-Hexadecanoic acid	256	$C_{16}H_{32}O_2$
SCA	15-Hydroxypentadecanoic acid	258	$C_{15}H_{30}O_3$
SCA	Octadecanoic acid	284	$C_{18}H_{36}O_2$
SCA	Azelaic acid	188	$C_9H_{16}O_4$
SCA	Tetradecanoic acid	228	$C_{14}H_{28}O_2$
SCA	12-Hydroxydodecanoic acid	216	$C_{12}H_{24}O_3$
SALK	Cycloeicosane	280	$C_{20}H_{40}$
SE	Hexadecanoic acid, 2-hydroxy-1-(hydroxymethyl)ethyl ester	330	$C_{19}H_{38}O_4$
BS	Bis(2-ethylhexyl) phthalate	390	$C_{24}H_{38}O_4$
USALC	2-Methyl-Z,Z-3,13-octadecadienol	280	$C_{19}H_{36}O$
USALC	12-Methyl-E,E-2,13-octadecadien-1-ol	280	$C_{19}H_{36}O$
USA	9,12-Octadecadienal	264	$C_{18}H_{32}O$
USA	2-Heptenal, (Z)-	112	$C_7H_{12}O$
USA	2-Decenal, (E)-	154	$C_{10}H_{18}O$
USA	2,4-Decadienal, (E,Z)-	152	$C_{10}H_{16}O$
USA	2,4-Decadienal, (E,E)-	152	$C_{10}H_{16}O$
USA	9-Octadecenal	266	$C_{18}H_{34}O$
USA	13-Tetradecenal	210	$C_{14}H_{26}O$
USA	9,17-Octadecadienal, (Z)-	264	$C_{18}H_{32}O$
USA	2-Decenal, (Z)-	154	$C_{10}H_{18}O$
USA	13-Octadecenal, (Z)-	266	$C_{18}H_{34}O$
USA	7-Hexadecenal, (Z)-	238	$C_{16}H_{30}O$
USA	cis-9-Hexadecenal	238	$C_{16}H_{30}O$
USA	9-Octadecenal, (Z)-	266	$C_{18}H_{34}O$
USA	2-Undecenal	168	$C_{11}H_{20}O$
USA	8-Hexadecenal, 14-methyl-, (Z)-	252	$C_{17}H_{32}O$
USA	2-Dodecenal	182	$C_{12}H_{22}O$
USA	cis-11-Hexadecenal	238	$C_{16}H_{30}O$
USCA	Oleic Acid	282	$C_{18}H_{34}O_2$

USCA	9,12-Octadecadienoic acid (Z,Z)-	280	$C_{18}H_{32}O_2$
USCA	Cyclopropaneoctanal, 2-octyl-	280	$C_{19}H_{36}O$
USCA	cis-Vaccenic acid	282	$C_{18}H_{34}O_2$
USCA	Palmitoleic acid	254	$C_{16}H_{30}O_2$
USCA	9-Eicosenoic acid, (Z)-	310	$C_{20}H_{38}O_2$
USCA	9-Octadecenoic acid (Z)-, 2-hydroxy-1-(hydroxymethyl)ethyl ester	356	$C_{21}H_{40}O_4$
USCA	trans-13-Octadecenoic acid	282	$C_{18}H_{34}O_2$
USCA	E-9-Tetradecenoic acid	226	$C_{14}H_{26}O_2$
USCA	Undecylenic acid	184	$C_{11}H_{20}O_2$
USCA	9-Oxononanoic acid	172	$C_9H_{16}O_3$
USCA	cis-7-Hexadecenoic acid	254	$C_{16}H_{30}O_2$
USCA	9-Hexadecenoic acid	254	$C_{16}H_{30}O_2$
USE	Glycidyl (Z)-9-Heptadecenoate	324	$C_{20}H_{36}O_3$
USE	9,12-Octadecadienoic acid (Z,Z)-, 2-hydroxy-1-(hydroxymethyl)ethyl ester	354	$C_{21}H_{38}O_4$
USE	9-Octadecenoic acid (Z)-, 2-hydroxyethyl ester	326	$C_{20}H_{38}O_3$
USE	1-cis-Vaccenoylglycerol	356	$C_{21}H_{40}O_4$
USE	Undec-10-ynoic acid, undecyl ester	336	$C_{22}H_{40}O_2$
USE	9-Octadecenoic acid (Z)-, 2,3-dihydroxypropyl ester	356	$C_{21}H_{40}O_4$
USE	9-Octadecenoic acid (Z)-, methyl ester	296	$C_{19}H_{36}O_2$
USE	Glycerol 1-palmitate	330	$C_{19}H_{38}O_4$
HGC	9,12-Octadecadienoyl chloride, (Z,Z)-	298	$C_{18}H_{31}ClO$
HGC	Oleyl alcohol, chlorodifluoroacetate	380	$C_{20}H_{35}ClF_2O_2$
HCC	9-Oxabicyclo[6.1.0]nonane	126	$C_8H_{14}O$
HCC	9-Oxabicyclo[6.1.0]nonane, cis-	126	$C_8H_{14}O$

Table S5 Different compounds in Northern (N) and Southern (S).

Spcies	Chemical	Molecular weight	Chemical formula	Region
SALC	Cyclohexanol, 3-methyl-	114	$C_7H_{14}O$	N
SALC	1,15-Pentadecanediol	244	$C_{15}H_{32}O_2$	N
SALC	Ethanol, 2-(2-butoxyethoxy)-	162	$C_8H_{18}O_3$	N
SALC	Behenic alcohol	326	$C_{22}H_{46}O$	S
SA	D-erythro-Pentose, 2-deoxy-	134	$C_5H_{10}O_4$	N
SA	Octadecanal	268	$C_{18}H_{36}O$	S
**SCA**	Pentanoic acid, 3-methyl-	116	$C_6H_{12}O_2$	S
**SCA**	Heptanoic acid	130	$C_7H_{14}O_2$	S
**SCA**	Heptadecanoic acid	270	$C_{17}H_{34}O_2$	S
**SCA**	Tridecanoic acid	214	$C_{13}H_{26}O_2$	S
**SCA**	Pentadecanoic acid	242	$C_{15}H_{30}O_2$	N
**SCA**	Dodecanal	184	$C_{12}H_{24}O$	N
SALK	Oxirane, hexyl-	128	$C_8H_{16}O$	S
SALK	13-Oxabicyclo[10.1.0]tridecane	182	$C_{12}H_{22}O$	S
SALK	Bicyclo[3.1.1]heptane, 2,6,6-trimethyl-, [1R-(1.alpha.,2.alpha.,5.alpha.)]-	138	$C_{10}H_{18}$	S
SALK	Cyclodecane	140	$C_{10}H_{20}$	N
SALK	Hexacosyl nonyl ether	509	$C_{35}H_{72}O$	N
SALK	Oxirane, 2-butyl-3-methyl-, cis-	114	$C_7H_{14}O$	N
SALK	Docosane, 7-butyl-	366	$C_{26}H_{54}$	N
SALK	1,2-15,16-Diepoxyhexadecane	254	$C_{16}H_{30}O_2$	N
SE	Tributyl acetylcitrate	402	$C_{20}H_{34}O_8$	S
SE	Glycidyl palmitate	312	$C_{19}H_{36}O_3$	S
SE	Octadecanoic acid, 2,3-dihydroxypropyl ester	358	$C_{21}H_{42}O_4$	S
SE	Hexadecanoic acid, ethyl ester	284	$C_{18}H_{36}O_2$	S
SE	Oxiraneundecanoic acid, 3-pentyl-, methyl ester, trans-	312	$C_{19}H_{36}O_3$	S
SE	Heptadecanoic acid, heptadecyl ester	509	$C_{34}H_{68}O_2$	N
SE	Butyric acid, 2-pentadecyl ester	298	$C_{19}H_{38}O_2$	N
BS	Estra-1,3,5(10)-trien-17.beta.-ol	256	$C_{18}H_{24}O$	S
BS	1,3-Benzenedicarboxylic acid, bis(2-ethylhexyl) ester	390	$C_{24}H_{38}O_4$	S
BS	1,4-Benzenedicarboxylic acid, bis(2-ethylhexyl) ester	390	$C_{24}H_{38}O_4$	S
BS	1,2-Benzenedicarboxylic acid, mononony1 ester	292	$C_{17}H_{24}O_4$	N
BS	1,2-Benzenedicarboxylic acid, bis(2-methylpropyl) ester	278	$C_{16}H_{22}O_4$	N
BS	Phthalic acid, butyl undecyl ester	376	$C_{23}H_{36}O_4$	N
BS	Phthalic acid, 5-methylhex-2-yl heptadecyl ester	502	$C_{32}H_{54}O_4$	N
BS	Phthalic acid, ethyl tetradecyl ester	390	$C_{24}H_{38}O_4$	N
BS	Phthalic acid, isobutyl octadecyl ester	474	$C_{30}H_{50}O_4$	N

USALC	E-2-Octadecadecen-1-ol	268	$C_{18}H_{36}O$	S
USALC	9,12-Octadecadien-1-ol, (Z,Z)-	266	$C_{18}H_{34}O$	S
USALC	Z,Z-3,13-Octadecadien-1-ol	266	$C_{18}H_{34}O$	S
USALC	Z,E-3,13-Octadecadien-1-ol	266	$C_{18}H_{34}O$	S
USALC	6,9,12-Octadecatrien-1-ol	264	$C_{18}H_{32}O$	N
USALC	9(E),11(E)-Conjugated linoleic acid	280	$C_{18}H_{32}O2$	S
USALC	2,4-Nonadienal, (E,E)-	138	$C_9H_{14}O$	S
USALC	2-Tridecenal, (E)-	196	$C_{13}H_{24}O$	S
USALC	cis,cis-7,10,-Hexadecadienal	236	$C_{16}H_{28}O$	S
USALC	2-Nonenal, (E)-	140	$C_9H_{16}O$	S
USALC	2,4-Decadienal	152	$C_{10}H_{16}O$	S
USALC	2-Octenal, (E)-	126	$C_8H_{14}O$	S
USALC	2,4-Dodecadienal, (E,E)-	180	$C_{12}H_{20}O$	S
USALC	9-Tetradecenal, (Z)-	210	$C_{14}H_{26}O$	S
USALC	2-Dodecenal, (E)-	182	$C_{12}H_{22}O$	N
USALC	2-Undecenal, E-	168	$C_{11}H_{20}O$	N
USALC	Oxalic acid, allyl dodecyl ester	298	$C_{17}H_{30}O_4$	N
USCA	Linoelaidic acid	280	$C_{18}H_{32}O_2$	S
USCA	17-Octadecynoic acid	280	$C_{18}H_{32}O_2$	S
USCA	Cyclopentaneundecanoic acid	254	$C_{16}H_{30}O_2$	S
USCA	2-Octenoic acid	142	$C_8H_{14}O_2$	S
USCA	E,E-10,12-Hexadecadien-1-ol acetate	280	$C_{18}H_{32}O_2$	S
USCA	2-Dodecenoic acid	198	$C_{12}H_{22}O_2$	S
USCA	9-Octadecenoic acid	282	$C_{18}H_{34}O_2$	S
USCA	trans-Traumatic acid	228	$C_{12}H_{20}O_4$	S
USCA	Z-7-Tetradecenoic acid	226	$C_{14}H_{26}O_2$	S
USCA	Z-8-Methyl-9-tetradecenoic acid	240	$C_{15}H_{28}O_2$	S
USCA	cis-13-Octadecenoic acid	282	$C_{18}H_{34}O_2$	S
USCA	2-Nonynoic acid	154	$C_9H_{14}O_2$	N
USE	Undec-10-ynoic acid, dodecyl ester	350	$C_{23}H_{42}O_2$	S
USE	Glutaric acid, tridec-2-yn-1-yl dec-4-enyl ester	448	$C_{28}H_{48}O_4$	S
USE	Decanoic acid, silver(1+) salt	278	$C_{10}H_{19}AgO_2$	S
USA	Pentanoic acid, 10-undecenyl ester	254	$C_{16}H_{30}O_2$	S
USA	Oxalic acid, cyclobutyl hexadecyl ester	368	$C_{22}H_{40}O_4$	S
USA	1,1-Dodecanediol, diacetate	286	$C_{16}H_{30}O_4$	N
USA	Glycidyl palmitoleate	310	$C_{19}H_{34}O_3$	N
USA	n-Propyl 11-octadecenoate	324	$C_{21}H_{40}O_2$	N
USA	Carbonic acid, decyl vinyl ester	228	$C_{13}H_{24}O_3$	N
USA	Undec-10-ynoic acid, tetradecyl ester	378	$C_{25}H_{46}O_2$	N
USA	Z-(13,14-Epoxy)tetradec-11-en-1-ol acetate	268	$C_{16}H_{28}O_3$	N
USA	trans-9-Octadecenoic acid, pentyl ester	352	$C_{23}H_{44}O_2$	N

USA	Methyl 7,8-octadecadienoate	294	$C_{19}H_{34}O_2$	N
HGC	Oleyl alcohol, trifluoroacetate	364	$C_{20}H_{35}F_3O_2$	S
HGC	Trichloroacetic acid, undec-2-enyl ester	314	$C_{13}H_{21}Cl_3O_2$	S
HGC	Trichloroacetic acid, undec-10-enyl ester	314	$C_{13}H_{21}Cl_3O_2$	S
HGC	Carbonic acid, 2,2,2-trichloroethyl undec-10-enyl ester	344	$C_{14}H_{23}C_{13}O_3$	S
HGC	(Z)-Tetradec-11-en-1-yl 2,2,2-trifluoroacetate	308	$C_{16}H_{27}F_3O_2$	S
HGC	Acetic acid, trifluoro-, dodecyl ester	282	$C_{14}H_{25}F_3O_2$	S
HGC	(E)-tetradec-11-en-1-yl 2,2,3,3,3-pentafluoropopanoate	358	$C_{17}H_{27}F_5O_2$	S
HGC	6-Methyl-2-heptanol, trifluoroacetate	226	$C_{10}H_{17}F_3O_2$	N
HGC	Octanoic acid, silver(1+) salt	250	$C_8H_{15}AgO_2$	N
HGC	4-Chloro-3-n-hexyltetrahydropyran	204	$C_{11}H_{21}C_lO$	N
HGC	(E)-Tetradec-11-en-1-yl 2,2,3,3,4,4,4-heptafluorobutanoate	408	$C_{18}H_{27}F_7O_2$	N
HGC	2- Chloropropionic acid, hexadecyl ester	332	$C_{19}H_{37}C_lO_2$	N
HGC	7-Heptadecene, 1-chloro-	272	$C_{17}H_{33}C_l$	N
OF	8-Heptadecene	238	$C_{17}H_{34}$	S
OF	Cyclodecene	138	$C_{10}H_{18}$	S
OF	1-Hexene, 4,5-dimethyl-	112	C_8H_{16}	S
OF	7-Tetradecenal, (Z)-	210	$C_{14}H_{26}O$	S
OF	1-Heptadecene	238	$C_{17}H_{34}$	S
OF	1-Eicosene	280	$C_{20}H_{40}$	S
OF	E,Z-1,3,12-Nonadecatriene	262	$C_{19}H_{34}$	N
OF	4-Tetradecene, (Z)-	196	$C_{14}H_{28}$	N
HCC	.alpha.-D-Mannopyranoside, methyl 3,6-anhydro-	176	$C_7H_{12}O_5$	S
HCC	2(1H)-Naphthalenone, octahydro-, trans-	152	$C_{10}H_{16}O$	S
HCC	Oxalic acid, cyclohexylmethyl octyl ester	298	$C_{17}H_{30}O_4$	S
HCC	Stevioside	804	$C_{38}H_{60}O_{18}$	S
HCC	2,5,6-Trimethyl-1,3-oxathiane	146	$C_7H_{14}OS$	S
HCC	2H-Pyran, 3,4-dihydro-	84	C_5H_8O	S
HCC	4-Cyclononen-1-one	138	$C_9H_{14}O$	S
HCC	9-Methylbicyclo[3.3.1]nonane	138	$C_{10}H_{18}$	S
HCC	Furan, 2-pentyl-	138	$C_9H_{14}O$	S
HCC	8-Oxabicyclo[5.1.0]octane	112	$C_7H_{12}O$	S
HCC	Cyclododecanone, 2-methylene-	194	$C_{13}H_{22}O$	S
HCC	cis-Dihydrocarvone	152	$C_{10}H_{16}O$	S
HCC	D-chiro-Inositol, 3-O-(2-amino-4-((carboxyiminomethyl)amino)-2,3,4,6-tetradeoxy-.alpha.-D-arabino-hexopyranosyl)-	379	$C_{14}H_{25}N_3O_9$	N
HCC	9-Borabicyclo[3.3.1]nonane, 9-hydroxy-	138	$C_8H_{15}BO$	N
HCC	Oxacyclohexadecan-2-one	240	$C_{15}H_{28}O_2$	N

HCC	1,6-Cyclodecadiene	136	$C_{10}H_{16}$	N

Table S6 Relative Content of Compounds in Northern and Southern Regions.

Region	site	SCA	SA	SE	USCA	USA	USE	USALC	BS	SALK	HGC	HCC	OF	SALC
Northeast	D1	7.05%	0.00%	5.04%	34.50%	8.94%	5.34%	0.00%	3.19%	0.74%	0.00%	0.00%	0.00%	0.58%
Northeast	D2	17.09%	0.00%	0.00%	32.42%	17.69%	0.07%	0.00%	3.31%	0.00%	0.00%	1.02%	0.00%	0.00%
Northeast	D3	7.09%	0.00%	0.00%	4.60%	2.28%	12.75%	34.28%	3.77%	0.25%	0.00%	0.00%	0.00%	0.00%
Northwest	X1	10.51%	5.92%	0.05%	39.04%	13.79%	2.62%	0.14%	1.52%	0.79%	2.36%	0.00%	0.00%	0.00%
Northwest	X2	18.64%	1.18%	2.52%	57.89%	1.08%	8.89%	0.00%	0.00%	0.04%	1.24%	0.00%	0.00%	0.00%
Northwest	X3	20.74%	1.26%	0.00%	45.05%	6.55%	5.16%	0.08%	0.00%	1.20%	0.13%	0.04%	0.00%	0.02%
North China	J1	4.37%	0.00%	0.00%	1.37%	8.49%	6.98%	46.47%	0.00%	0.00%	0.04%	0.32%	0.14%	1.09%
North China	J2	4.01%	0.38%	2.79%	39.21%	17.47%	12.34%	0.06%	1.08%	0.00%	0.41%	1.02%	0.24%	0.00%
North China	J3	3.73%	0.00%	0.00%	0.20%	74.31%	13.36%	0.06%	0.00%	0.06%	0.09%	1.10%	0.00%	0.00%
Southeast	M1	35.04%	0.14%	11.50%	28.89%	0.00%	9.78%	0.00%	0.00%	0.00%	0.00%	0.00%	0.00%	0.00%
Southeast	M2	47.27%	0.69%	0.00%	19.85%	1.38%	0.00%	0.00%	1.39%	0.00%	0.00%	0.63%	0.00%	0.00%
Southeast	M3	17.36%	3.01%	2.72%	16.86%	9.89%	0.00%	0.00%	9.73%	0.00%	0.00%	0.14%	0.00%	0.00%
Southeast	S1	18.86%	0.24%	0.00%	60.75%	0.16%	2.89%	0.00%	0.00%	0.00%	0.23%	0.66%	0.00%	0.00%
Southeast	S2	1.73%	3.84%	8.99%	39.96%	23.10%	0.66%	2.66%	0.00%	0.17%	0.31%	0.48%	0.94%	0.00%
Southeast	S3	7.22%	3.03%	0.00%	2.84%	54.29%	0.20%	0.12%	0.00%	0.00%	0.00%	1.57%	5.83%	0.37%
Southwest	G1	14.73%	0.22%	0.00%	82.85%	0.25%	0.12%	1.49%	0.00%	0.00%	0.00%	0.00%	0.01%	0.00%
Southwest	G2	5.13%	0.15%	6.90%	59.92%	12.71%	0.00%	0.20%	0.00%	0.00%	4.26%	0.94%	0.00%	0.00%
Southwest	G3	9.18%	0.09%	0.00%	54.03%	15.18%	0.16%	0.11%	0.00%	0.00%	0.00%	0.00%	0.00%	0.55%
Southwest	C1	9.21%	3.97%	0.00%	42.10%	12.65%	0.63%	0.12%	5.01%	0.98%	0.69%	0.15%	0.00%	0.00%
Southwest	C2	5.71%	0.04%	0.04%	66.92%	2.16%	11.03%	0.19%	0.00%	0.19%	0.05%	0.33%	0.14%	0.00%
Southwest	C3	4.73%	2.32%	5.63%	43.85%	16.38%	0.39%	0.08%	11.70%	0.00%	0.00%	4.04%	0.06%	0.00%

Table S7 The same compounds in southeast (M-region+S-region) and southwest (C-region + G-region).

Spcies	Chemical	Molecular weight	Chemical formula
SA	Octanal	128	$C_8H_{16}O$
SA	Nonanal	142	$C_9H_{18}O$
SCA	Hexanoic acid	116	$C_6H_{12}O_2$
SCA	Octanoic acid	144	$C_8H_{16}O_2$
SCA	Nonanoic acid	158	$C_9H_{18}O_2$
SCA	n-Hexadecanoic acid	256	$C_{16}H_{32}O_2$
SCA	Heptanoic acid	130	$C_7H_{14}O_2$
SCA	Azelaic acid	188	$C_9H_{16}O_4$
SCA	Tetradecanoic acid	228	$C_{14}H_{28}O_2$
SE	Hexadecanoic acid, 2-hydroxy-1-(hydroxymethyl)ethyl ester	330	$C_{19}H_{38}O_4$
BS	Bis(2-ethylhexyl) phthalate	390	$C_{24}H_{38}O_4$
USALC	E-2-Octadecadecen-1-ol	268	$C_{18}H_{36}O$
USALC	2-Methyl-Z,Z-3,13-octadecadienol	280	$C_{19}H_{36}O$
USA	9,12-Octadecadienal	264	C18H32O
USA	9(E),11(E)-Conjugated linoleic acid	280	$C_{18}H_{32}O_2$
USA	2,4-Decadienal, (E,Z)-	152	$C_{10}H_{16}O$
USA	2,4-Decadienal, (E,E)-	152	$C_{10}H_{16}O$
USA	2-Tridecenal, (E)-	196	$C_{13}H_{24}O$
USA	9-Octadecenal	266	$C_{18}H_{34}O$
USA	9,17-Octadecadienal, (Z)-	264	$C_{18}H_{32}O$
USA	13-Octadecenal, (Z)-	266	$C_{18}H_{34}O$
USA	cis-9-Hexadecenal	238	$C_{16}H_{30}O$
USA	9-Octadecenal, (Z)-	266	$C_{18}H_{34}O$
USCA	Oleic Acid	282	$C1_8H_{34}O2$
USCA	9,12-Octadecadienoic acid (Z,Z)-	280	$C_{18}H_{32}O_2$
USCA	Linoelaidic acid	280	$C_{18}H_{32}O_2$
USCA	Cyclopropaneoctanal, 2-octyl-	280	$C_{19}H_{36}O$
USCA	17-Octadecynoic acid	280	$C_{18}H_{32}O_2$
USCA	cis-Vaccenic acid	282	$C_{18}H_{34}O_2$
USCA	Palmitoleic acid	254	$C_{16}H_{30}O_2$
USCA	Cyclopentaneundecanoic acid	254	$C_{16}H_{30}O_2$
USCA	9-Eicosenoic acid, (Z)-	310	$C_{20}H_{38}O_2$

USCA	9-Octadecenoic acid (Z)-, 2-hydroxy-1-(hydroxymethyl)ethyl ester	356	$C_{21}H_{40}O_4$
USCA	trans-13-Octadecenoic acid	282	$C_{18}H_{34}O_2$
USE	Glycidyl (Z)-9-Heptadecenoate	324	$C_{20}H_{36}O_3$
USE	9-Octadecenoic acid (Z)-, 2-hydroxyethyl ester	326	$C_{20}H_{38}O_3$
USE	Undec-10-ynoic acid, undecyl ester	336	$C_{22}H_{40}O_2$
USE	Undec-10-ynoic acid, dodecyl ester	350	$C_{23}H_{42}O_2$
HGC	9,12-Octadecadienoyl chloride, (Z,Z)-	298	$C_{18}H_{31}C_lO$
HGC	Oleyl alcohol, chlorodifluoroacetate	380	$C_{20}H_{35}C_lF_2O_2$
HGC	Trichloroacetic acid, undec-10-enyl ester	314	$C_{13}H_{21}C_{l3}O_2$
HCC	.alpha.-D-Mannopyranoside, methyl 3,6-anhydro-	176	$C_7H_{12}O_5$
HCC	9-Oxabicyclo[6.1.0]nonane	126	$C_8H_{14}O$
HCC	9-Oxabicyclo[6.1.0]nonane, cis-	126	$C_8H_{14}O$
HCC	Stevioside	804	$C_{38}H_{60}O_{18}$

Table S8 The different compounds in southeast (M-region+S-region) and southwest (C-region + G-region).

Spcies	Chemical	Molecular weight	Chemical formula	Region
SALC	Decanal	156	$C_{10}H_{20}O$	sE
SALC	Behenic alcohol	326	$C_{22}H_{46}O$	sW
SA	D-erythro-Pentose, 2-deoxy-	134	$C_5H_{10}O_4$	sE
SA	Octadecanal	268	$C_{18}H_{36}O$	sW
SCA	Pentanoic acid, 3-methyl-	116	$C_6H_{12}O_2$	sE
SCA	15-Hydroxypentadecanoic acid	258	$C_{15}H_{30}O_3$	sE
SCA	Octadecanoic acid	284	$C_{18}H_{36}O_2$	sE
SCA	Heptadecanoic acid	270	$C_{17}H_{34}O_2$	sE
SCA	12-Hydroxydodecanoic acid	216	$C_{12}H_{24}O_3$	sW
SCA	Tridecanoic acid	214	$C_{13}H_{26}O_2$	sW
SALK	Oxirane, hexyl-	128	$C_8H_{16}O$	sE
SALK	13-Oxabicyclo[10.1.0]tridecane	182	$C_{12}H_{22}O$	sE
SALK	Cycloeicosane	280	$C_{20}H_{40}$	sW
SALK	Bicyclo[3.1.1]heptane, 2,6,6-trimethyl-, [1R-(1.alpha.,2.alpha.,5.alpha.)]-	138	$C_{10}H_{18}$	sW
SE	Tributyl acetylcitrate	402	$C_{20}H_{34}O_8$	sE
SE	Glycidyl palmitate	312	$C_{19}H_{36}O_3$	sE
SE	Octadecanoic acid, 2,3-dihydroxypropyl ester	358	$C_{21}H_{42}O_4$	sE
SE	Hexadecanoic acid, ethyl ester	284	$C_{18}H_{36}O_2$	sW
SE	Oxiraneundecanoic acid, 3-pentyl-, methyl ester, trans-	312	$C_{19}H_{36}O_3$	sW
BS	Estra-1,3,5(10)-trien-17.beta.-ol	256	$C_{18}H_{24}O$	sE
BS	1,3-Benzenedicarboxylic acid, bis(2-ethylhexyl) ester	390	$C_{24}H_{38}O_4$	sE
BS	1,4-Benzenedicarboxylic acid, bis(2-ethylhexyl) ester	390	$C_{24}H_{38}O_4$	sW
USALC	9,12-Octadecadien-1-ol, (Z,Z)-	266	$C_{18}H_{34}O$	sE
USALC	12-Methyl-E,E-2,13-octadecadien-1-ol	280	$C_{19}H_{36}O$	sW
USALC	Z,Z-3,13-Octadecadien-1-ol	266	$C_{18}H_{34}O$	sW
USALC	Z,E-3,13-Octadecadien-1-ol	266	$C_{18}H_{34}O$	sW
USA	2-Heptenal, (Z)-	112	$C_7H_{12}O$	sE
USA	2,4-Nonadienal, (E,E)-	138	$C_9H_{14}O$	sE
USA	2-Decenal, (E)-	154	$C_{10}H_{18}O$	sE
USA	13-Tetradecenal	210	$C_{14}H_{26}O$	sE
USA	cis,cis-7,10,-Hexadecadienal	236	$C_{16}H_{28}O$	sE
USA	2-Decenal, (Z)-	154	$C_{10}H_{18}O$	sE

USA	7-Hexadecenal, (Z)-	238	$C_{16}H_{30}O$	sE
USA	2-Nonenal, (E)-	140	$C_9H_{16}O$	sE
USA	2,4-Decadienal	152	$C_{10}H_{16}O$	sE
USA	2-Undecenal	168	$C_{11}H_{20}O$	sE
USA	8-Hexadecenal, 14-methyl-, (Z)-	252	$C_{17}H_{32}O$	sW
USA	2-Dodecenal	182	$C_{12}H_{22}O$	sW
USA	cis-11-Hexadecenal	238	$C_{16}H_{30}O$	sW
USA	2-Octenal, (E)-	126	$C_8H_{14}O$	sW
USA	2,4-Dodecadienal, (E,E)-	180	$C_{12}H_{20}O$	sW
USA	9-Tetradecenal, (Z)-	210	$C_{14}H_{26}O$	sW
USCA	2-Octenoic acid	142	$C_8H_{14}O_2$	sE
USCA	E,E-10,12-Hexadecadien-1-ol acetate	280	$C_{18}H_{32}O_2$	sE
USCA	2-Dodecenoic acid	198	$C_{12}H_{22}O_2$	sE
USCA	E-9-Tetradecenoic acid	226	$C_{14}H_{26}O_2$	sW
USCA	Undecylenic acid	184	$C_{11}H_{20}O_2$	sW
USCA	9-Oxononanoic acid	172	$C_9H_{16}O_3$	sW
USCA	cis-7-Hexadecenoic acid	254	$C_{16}H_{30}O_2$	sW
USCA	9-Hexadecenoic acid	254	$C_{16}H_{30}O_2$	sW
USCA	9-Octadecenoic acid	282	$C_{18}H_{34}O_2$	sW
USCA	trans-Traumatic acid	228	$C_{12}H_{20}O_4$	sW
USCA	Z-7-Tetradecenoic acid	226	$C_{14}H_{26}O_2$	sW
USCA	Z-8-Methyl-9-tetradecenoic acid	240	$C_{15}H_{28}O_2$	sW
USCA	cis-13-Octadecenoic acid	282	$C_{18}H_{34}O_2$	sW
USE	9,12-Octadecadienoic acid (Z,Z)-, 2-hydroxy-1-(hydroxymethyl)ethyl ester	354	$C_{21}H_{38}O_4$	sE
USE	1-cis-Vaccenoylglycerol	356	$C_{21}H_{40}O_4$	sE
USE	Glutaric acid, tridec-2-yn-1-yl dec-4-enyl ester	448	$C_{28}H_{48}O_4$	sE
USE	9-Octadecenoic acid (Z)-, 2,3-dihydroxypropyl ester	356	$C_{21}H_{40}O_4$	sW
USE	9-Octadecenoic acid (Z)-, methyl ester	296	$C_{19}H_{36}O_2$	sW
USE	Glycerol 1-palmitate	330	$C_{19}H_{38}O_4$	sW
USE	Decanoic acid, silver(1+) salt	278	$C_{10}H_{19}AgO_2$	sW
USE	Pentanoic acid, 10-undecenyl ester	254	$C_{16}H_{30}O_2$	sW
USE	Oxalic acid, cyclobutyl hexadecyl ester	368	$C_{22}H_{40}O_4$	sW
HGC	Oleyl alcohol, trifluoroacetate	364	$C_{20}H_{35}F_3O_2$	sE
HGC	Trichloroacetic acid, undec-2-enyl ester	314	$C_{13}H_{21}Cl_3O_2$	sE
HGC	Carbonic acid, 2,2,2-trichloroethyl undec-10-enyl ester	344	$C_{14}H_{23}Cl_3O_3$	sE
HGC	(Z)-Tetradec-11-en-1-yl 2,2,2-trifluoroacetate	308	$C_{16}H_{27}F_3O_2$	sE

HGC	Acetic acid, trifluoro-, dodecyl ester	282	$C_{14}H_{25}F_3O_2$	sW
HGC	(E)-tetradec-11-en-1-yl 2,2,3,3,3-pentafluoropopanoate	358	$C_{17}H_{27}F_5O_2$	sW
OF	8-Heptadecene	238	$C_{17}H_{34}$	sE
OF	Cyclodecene	138	$C_{10}H_{18}$	sE
OF	1-Hexene, 4,5-dimethyl-	112	C_8H_{16}	sE
OF	7-Tetradecenal, (Z)-	210	$C_{14}H_{26}O$	sE
OF	1-Heptadecene	238	$C_{17}H_{34}$	sW
OF	1-Eicosene	280	$C_{20}H_{40}$	sW
HCC	2(1H)-Naphthalenone, octahydro-, trans-	152	$C_{10}H_{16}O$	sE
HCC	Oxalic acid, cyclohexylmethyl octyl ester	298	$C_{17}H_{30}O_4$	sE
HCC	2,5,6-Trimethyl-1,3-oxathiane	146	$C_7H_{14}OS$	sE
HCC	2H-Pyran, 3,4-dihydro-	84	C_5H_8O	sE
HCC	4-Cyclononen-1-one	138	$C_9H_{14}O$	sE
HCC	9-Methylbicyclo[3.3.1]nonane	138	$C_{10}H_{18}$	sE
HCC	Furan, 2-pentyl-	138	$C_9H_{14}O$	sE
HCC	8-Oxabicyclo[5.1.0]octane	112	$C_7H_{12}O$	sW
HCC	Cyclododecanone, 2-methylene-	194	$C_{13}H_{22}O$	sW
HCC	cis-Dihydrocarvone	152	$C_{10}H_{16}O$	sW

"sW ", Southwest; "sE ", Southeast.

Table S9 Compounds in Northern.

	Spcies	Chemical	Molecular weight	D	X	J
SALC	Decanal	156	$C_{10}H_{20}O$		√	
SALC	Cyclohexanol, 3-methyl-	114	$C_7H_{14}O$	√		
SALC	1,15-Pentadecanediol	244	$C_{15}H_{32}O_2$		√	
SALC	Ethanol, 2-(2-butoxyethoxy)-	162	$C_8H_{18}O_3$			√
SA	Octanal	128	$C_8H_{16}O$		√	
SA	Nonanal	142	$C_9H_{18}O$		√	
SA	D-erythro-Pentose, 2-deoxy-	134	$C_5H_{10}O_4$			√
SCA	Hexanoic acid	116	$C_6H_{12}O_2$	√	√	√
SCA	Octanoic acid	144	$C_8H_{16}O_2$	√	√	
SCA	Nonanoic acid	158	$C_9H_{18}O_2$		√	
SCA	n-Hexadecanoic acid	256	$C_{16}H_{32}O_2$	√	√	√
SCA	15-Hydroxypentadecanoic acid	258	$C_{15}H_{30}O_3$	√	√	
SCA	Octadecanoic acid	284	$C_{18}H_{36}O_2$	√	√	
SCA	Azelaic acid	188	$C_9H_{16}O_4$		√	
SCA	Tetradecanoic acid	228	$C_{14}H_{28}O_2$		√	
SCA	12-Hydroxydodecanoic acid	216	$C_{12}H_{24}O_3$	√	√	
SCA	Pentadecanoic acid	242	$C_{15}H_{30}O_2$		√	

SCA	Dodecanal	184	$C_{12}H_{24}O$		√	
SAL K	Cyclodecane	140	$C_{10}H_{20}$	√		
SAL K	Hexacosyl nonyl ether	509	$C_{35}H_{72}O$	√		
SAL K	Oxirane, 2-butyl-3-methyl-, cis-	114	$C_{7}H_{14}O$		√	
SAL K	Docosane, 7-butyl-	366	$C_{26}H_{54}$		√	
SAL K	Cycloeicosane	280	$C_{20}H_{40}$		√	
SAL K	1,2-15,16-Diepoxyhexadecane	254	$C_{16}H_{30}O_{2}$			√
SE	Hexadecanoic acid, 2-hydroxy-1-(hydroxymethyl)ethyl ester	330	$C_{19}H_{38}O_{4}$	√	√	√
SE	Heptadecanoic acid, heptadecyl ester	509	$C_{34}H_{68}O_{2}$		√	
SE	Butyric acid, 2-pentadecyl ester	298	$C_{19}H_{38}O_{2}$			√
BS	Bis(2-ethylhexyl) phthalate	390	$C_{24}H_{38}O_{4}$	√		
BS	1,2-Benzenedicarboxylic acid, mononony l ester	292	$C_{17}H_{24}O_{4}$	√		
BS	1,2-Benzenedicarboxylic acid, bis(2-methylpropyl) ester	278	$C_{16}H_{22}O_{4}$	√		
BS	Phthalic acid, butyl undecyl ester	376	$C_{23}H_{36}O_{4}$	√		
BS	Phthalic acid, 5-methylhex-2-yl heptadecyl ester	502	$C_{32}H_{54}O_{4}$	√		
BS	Phthalic acid, ethyl tetradecyl ester	390	$C_{24}H_{38}O_{4}$		√	
BS	Phthalic acid, isobutyl octadecyl ester	474	$C_{30}H_{50}O_{4}$			√
USA	2-Methyl-Z,Z-3,13-octadecadienol	280	$C_{19}H_{36}O$	√	√	√

LC						
USA<br>LC	12-Methyl-E,E-2,13-octadecadien-1-ol	280	$C_{19}H_{36}O$		√	
USA<br>LC	6,9,12-Octadecatrien-1-ol	264	$C_{18}H_{32}O$			√
USA	9,12-Octadecadienal	264	$C_{18}H_{32}O$	√	√	√
USA	2-Heptenal, (Z)-	112	$C_7H_{12}O$	√		
USA	2-Decenal, (E)-	154	$C_{10}H_{18}O$		√	√
USA	2,4-Decadienal, (E,Z)-	152	$C_{10}H_{16}O$	√	√	√
USA	2,4-Decadienal, (E,E)-	152	$C_{10}H_{16}O$	√	√	√
USA	9-Octadecenal	266	$C_{18}H_{34}O$		√	
USA	13-Tetradecenal	210	$C_{14}H_{26}O$			√
USA	9,17-Octadecadienal, (Z)-	264	$C_{18}H_{32}O$		√	√
USA	2-Decenal, (Z)-	154	$C_{10}H_{18}O$	√	√	
USA	13-Octadecenal, (Z)-	266	$C_{18}H_{34}O$	√		√
USA	7-Hexadecenal, (Z)-	238	$C_{16}H_{30}O$		√	
USA	cis-9-Hexadecenal	238	$C_{16}H_{30}O$			√
USA	9-Octadecenal, (Z)-	266	$C_{18}H_{34}O$		√	√
USA	2-Undecenal	168	$C_{11}H_{20}O$		√	
USA	8-Hexadecenal, 14-methyl-, (Z)-	252	$C_{17}H_{32}O$	√		
USA	2-Dodecenal, (E)-	182	$C_{12}H_{22}O$		√	
USA	2-Dodecenal	182	$C_{12}H_{22}O$		√	
USA	2-Undecenal, E-	168	$C_{11}H_{20}O$			√
USA	Oxalic acid, allyl dodecyl ester	298	$C_{17}H_{30}O_4$			√

USA	cis-11-Hexadecenal	238	$C_{16}H_{30}O$			√
USCA	Oleic Acid	282	$C_{18}H_{34}O_2$	√	√	√
USCA	9,12-Octadecadienoic acid (Z,Z)-	280	$C_{18}H_{32}O_2$		√	√
USCA	Cyclopropaneoctanal, 2-octyl-	280	$C_{19}H_{36}O$			√
USCA	cis-Vaccenic acid	282	$C_{18}H_{34}O_2$		√	
USCA	Palmitoleic acid	254	$C_{16}H_{30}O_2$		√	√
USCA	9-Eicosenoic acid, (Z)-	310	$C_{20}H_{38}O_2$		√	√
USCA	9-Octadecenoic acid (Z)-, 2-hydroxy-1-(hydroxymethyl)ethyl ester	356	$C_{21}H_{40}O_4$	√	√	√
USCA	trans-13-Octadecenoic acid	282	$C_{18}H_{34}O_2$		√	
USCA	E-9-Tetradecenoic acid	226	$C_{14}H_{26}O_2$	√		
USCA	Undecylenic acid	184	$C_{11}H_{20}O_2$		√	
USCA	9-Oxononanoic acid	172	$C_9H_{16}O_3$		√	
USCA	2-Nonynoic acid	154	$C_9H_{14}O_2$		√	

USC A	cis-7-Hexadecenoic acid	254	$C_{16}H_{30}O_2$			√
USC A	9-Hexadecenoic acid	254	$C_{16}H_{30}O_2$			√
USE	Glycidyl (Z)-9-Heptadecenoate	324	$C_{20}H_{36}O_3$		√	√
USE	9,12-Octadecadienoic acid (Z,Z)-, 2-hydroxy-1-(hydroxymethyl)ethyl ester	354	$C_{21}H_{38}O_4$			√
USE	9-Octadecenoic acid (Z)-, 2-hydroxyethyl ester	326	$C_{20}H_{38}O_3$		√	
USE	1-cis-Vaccenoylglycerol	356	$C_{21}H_{40}O_4$		√	√
USE	Undec-10-ynoic acid, undecyl ester	336	$C_{22}H_{40}O_2$		√	√
USE	9-Octadecenoic acid (Z)-, 2,3-dihydroxypropyl ester	356	$C_{21}H_{40}O_4$	√		√
USE	1,1-Dodecanediol, diacetate	286	$C_{16}H_{30}O_4$	√		
USE	Glycidyl palmitoleate	310	$C_{19}H_{34}O_3$	√		√
USE	n-Propyl 11-octadecenoate	324	$C_{21}H_{40}O_2$	√		
USE	Carbonic acid, decyl vinyl ester	228	$C_{13}H_{24}O_3$		√	
USE	Undec-10-ynoic acid, tetradecyl ester	378	$C_{25}H_{46}O_2$		√	
USE	9-Octadecenoic acid (Z)-, methyl ester	296	$C_{19}H_{36}O_2$		√	
USE	Z-(13,14-Epoxy)tetradec-11-en-1-ol acetate	268	$C_{16}H_{28}O_3$		√	√
USE	Glycerol 1-palmitate	330	$C_{19}H_{38}O_4$		√	
USE	trans-9-Octadecenoic acid, pentyl ester	352	$C_{23}H_{44}O_2$			√
USE	Methyl 7,8-octadecadienoate	294	$C_{19}H_{34}O_2$			√
HGC	6-Methyl-2-heptanol, trifluoroacetate	226	$C_{10}H_{17}F_3O_2$		√	
HGC	9,12-Octadecadienoyl chloride, (Z,Z)-	298	$C_{18}H_{31}ClO$			√
HGC	Oleyl alcohol, chlorodifluoroacetate	380	$C_{20}H_{35}ClF_2O_2$			√

HGC	Octanoic acid, silver(1+) salt	250	$C_8H_{15}AgO_2$		√	
HGC	4-Chloro-3-n-hexyltetrahydropyran	204	$C_{11}H_{21}ClO$		√	
HGC	(E)-Tetradec-11-en-1-yl 2,2,3,3,4,4,4-heptafluorobutanoate	408	$C_{18}H_{27}F_7O_2$		√	
HGC	2- Chloropropionic acid, hexadecyl ester	332	$C_{19}H_{37}ClO_2$			√
HGC	7-Heptadecene, 1-chloro-	272	$C_{17}H_{33}Cl$			√
OF	E,Z-1,3,12-Nonadecatriene	262	$C_{19}H_{34}$			√
OF	4-Tetradecene, (Z)-	196	$C_{14}H_{28}$			√
HCC	9-Oxabicyclo[6.1.0]nonane	126	$C_8H_{14}O$		√	√
HCC	9-Oxabicyclo[6.1.0]nonane, cis-	126	$C_8H_{14}O$			√
HCC	D-chiro-Inositol, 3-O-(2-amino-4-((carboxyiminomethyl)amino)-2,3,4,6-tetradeoxy-.alpha.-D-arabino-hexopyranosyl)-	379	$C_{14}H_{25}N_3O_9$	√		
HCC	9-Borabicyclo[3.3.1]nonane, 9-hydroxy-	138	$C_8H_{15}BO$		√	
HCC	Oxacyclohexadecan-2-one	240	$C_{15}H_{28}O_2$			√
HCC	1,6-Cyclodecadiene	136	$C_{10}H_{16}$			√

"D", D-region; "X", X-region; "J", J-region; "√", Compound contained in COF of certain region.
